# Network of biomarkers and their mediation effects on the associations between regular exercise and the incidence of cardiovascular & metabolic diseases

**DOI:** 10.1038/s41598-021-92312-x

**Published:** 2021-06-17

**Authors:** JooYong Park, Jaesung Choi, Ji-Eun Kim, Miyoung Lee, Aesun Shin, Jong-koo Lee, Daehee Kang, Ji-Yeob Choi

**Affiliations:** 1 Department of Biomedical Sciences, Seoul National University Graduate School, 103 Daehak-ro, Jongno-gu, Seoul, 03080 Korea; 2 Institute of Health Policy and Management, Seoul National University Medical Research Center, Seoul, Korea; 3 College of Physical Education and Sport Science, Kookmin University, Seoul, Korea; 4 Department of Preventive Medicine, Seoul National University College of Medicine, Seoul, Korea; 5 Cancer Research Institute, Seoul National University, Seoul, Korea; 6 JW Lee Center for Global Medicine, Seoul National University College of Medicine, Seoul, Korea; 7 Department of Family Medicine, Seoul National University College of Medicine, Seoul, Korea

**Keywords:** Biomarkers, Diseases, Cardiovascular diseases, Endocrine system and metabolic diseases, Risk factors, Disease prevention, Preventive medicine, Epidemiology

## Abstract

This study aimed to understand the biological process related to the prevention of cardiovascular & metabolic diseases (CMD), including diabetes, hypertension, and dyslipidemia via regular exercise. This study included 17,053 subjects aged 40–69 years in the Health Examinees Study from 2004 to 2012. Participation in regular exercise was investigated by questionnaires. Data on 42 biomarkers were collected from anthropometric measures and laboratory tests. We examined the associations between regular exercise and biomarkers using general linear models, between biomarkers and the risk of CMD using cox proportional hazard models, and the mediation effect of biomarkers using mediation analyses. Biomarker networks were constructed based on the significant differential correlations (*p* < 0.05) between the exercise and non-exercise groups in men and women, respectively. We observed significant mediators in 14 and 16 of the biomarkers in men and women, respectively. Triglyceride level was a noteworthy mediator in decreasing the risk of CMD with exercise, explaining 23.79% in men and 58.20% in women. The biomarker network showed comprehensive relationships and associations among exercise, biomarkers, and CMD. Body composition-related biomarkers were likely to play major roles in men, while obesity-related biomarkers seemed to be key factors in women.

## Introduction

Physical activity is a modifiable lifestyle factor with health benefits and preventive effects against many chronic diseases^1–3^. However, the mechanisms by which physical activity benefits health remain unclear^4^. To better understand these biological processes, comprehensive approaches are needed to evaluate biological factors and observe their changes. In this aspect, biomarkers might play a key role in revealing and explaining the biological processes by which exercise provides health benefits.

Most biomarkers are changed with aging^5^. Age is the most well-known risk factor for various diseases; thus, many studies have established biomarkers according to the criteria from American Federation of Aging Research to better understand the disease process with aging^6–9^. These biomarkers include not only molecular/DNA biomarkers such as telomere length but also blood-based clinical biomarkers and anthropometric measurements^7–9^ that are widely used to screen or to diagnose health conditions. Therefore, these biomarkers can be used to assist researchers in better understanding the biological processes during exercise and how exercise affects age-related diseases.

However, the traditional approach in epidemiology which is based on the regression models could examine only the associations between an exposure and an outcome. Therefore that approach could not explain complex relationships^10,11^. To uncover complicated relationships in the biological mechanisms, a wide variety of biomarkers should be available and comprehensive analysis should be needed in the context of systems epidemiology^12^. A network approach can help conduct integrative analysis and infer in the context of biological processes the relationship between the variables by nodes and edges^13,14^.

In this study, we hypothesized that regular exercise would have a beneficial effect on each biomarker related to the prevention of cardiovascular & metabolic diseases (CMD), including diabetes, hypertension, and dyslipidemia, and would ultimately lead to healthy aging. We used various biomarkers from the Health Examinees (HEXA) study to understand biological processes related to CMD prevention in middle-aged adults via regular exercise. We constructed biomarker networks to show the comprehensive relationships among various biomarkers and their associations with regular exercise and CMD.

## Results

### Characteristics of the study population

The basic characteristics of the included dataset (n = 17,053) differed from those of the excluded dataset (n = 45,098); however, a noteworthy imbalance was not observed in the evaluation of the standardized differences except for income (Supplementary Table S1). Age at baseline which ranged from 40 to 69 years was correlated with most of the biomarkers in both men and women (Supplementary Table S2). All analyses were performed in men and women separately because they differed in basic characteristics (Supplementary Table S3) and distribution of biomarkers at baseline (Supplementary Table S4; all *p* values < 00001, not shown). However, men and women had similar patterns of regular exercise; who were older, more educated, with a higher income, were unemployed or housewives, had never smoked, or currently drunk alcohol were more likely to participate in regular exercise (Table 1).

**Table 1:** Characteristics of the study population at baseline according to participation in regular exercise.

	Men				Women				p for heterogeneity
	Participation in regular exercise				Participation in regular exercise				
	No (reference) N = 2154 (40.0%)	Yes N = 3236 (60.0%)			No (reference) N = 5431 (46.6%)	Yes N = 6232 (53.4%)			
	%	%	OR^a^	(95% CI)	%	%	OR^a^	(95% CI)	
**Age, Mean ± SD (years)**	52.9 ± 8.40	54.8 ± 8.01			51.4 ± 7.72	52.4 ± 7.15			
40–44	22.6	13.9	1.00	(Reference)	23.8	15.8	1.00	(Reference)	
45–49	13.7	12.1	1.40	(1.14–1.72)	17.4	19.3	1.78	(1.57–2.02)	0.0514
50–54	19.2	20.5	1.82	(1.51–2.19)	24.6	27.2	1.83	(1.63–2.06)	0.9610
55–59	18.8	21.8	2.00	(1.65–2.43)	17.0	20.1	2.07	(1.81–2.36)	0.7740
60–64	16.6	18.9	1.90	(1.54–2.33)	11.7	12.3	1.87	(1.60–2.17)	0.9017
65–69	9.2	12.9	2.23	(1.74–2.85)	5.5	5.3	1.80	(1.48–2.19)	0.1813
**Education**									
≤ Middle school	23.7	16.5	1.00	(Reference)	32.7	30.6	1.00	(Reference)	
High school	42.3	38.3	1.40	(1.19–1.64)	44.6	47.8	1.17	(1.07–1.29)	0.0585
≥ College	33.4	45.1	1.74	(1.44–2.10)	22.3	21.3	1.15	(1.01–1.31)	0.0004
Unknown	0.3	0.2			0.3	0.3			
**Income (₩10,000)**									
< 200	25.3	18.7	1.00	(Reference)	27.9	22.6	1.00	(Reference)	
200–400	45.2	43.3	1.38	(1.18–1.62)	43.0	44.4	1.32	(1.19–1.47)	0.6519
≥ 400	27.1	36.2	1.71	(1.42–2.06)	26.3	31.3	1.62	(1.44–1.83)	0.6340
Unknown	2.5	1.9			2.8	1.7			
**Marital status**									
Living with spouse	92.6	94.8	1.00	(Reference)	87.3	90.1	1.00	(Reference)	
Living alone	7.2	5.2	1.03	(0.80–1.31)	12.6	9.9	1.02	(0.90–1.16)	0.9441
Unknown	0.2	0.1			0.1	0.1			
**Current occupation**									
Office	31.9	39.2	1.00	(Reference)	18.6	14.4	1.00	(Reference)	
Manual	54.7	38.4	0.76	(0.66–0.87)	33.2	21.2	0.84	(0.74–0.96)	0.3019
Unemployed/House wives	11.9	20.9	1.64	(1.34–2.01)	47.5	63.8	1.82	(1.62–2.05)	0.3865
Soldier/etc	1.2	1.3	0.97	(0.58–1.63)	0.4	0.4	1.04	(0.57–1.89)	0.8630
Unknown	0.3	0.2			0.3	0.3			
**BMI, kg/m**^**2**^									
< 18.5	1.5	1.0	0.73	(0.43–1.22)	3.0	2.0	0.66	(0.52–0.85)	0.7300
18.5–23	31.4	28.8	1.00	(Reference)	44.5	46.6	1.00	(Reference)	
23–25	30.8	31.2	1.05	(0.91–1.22)	24.8	27.1	1.02	(0.93–1.12)	0.7480
25–30	33.4	36.9	1.17	(1.01–1.35)	24.8	22.6	0.87	(0.79–0.96)	0.0008
≥ 30	2.8	2.1	0.87	(0.60–1.26)	2.9	1.8	0.61	(0.47–0.79)	0.1234
**Smoking**									
Never	26.7	33.6		(Reference)	96.9	97.9	1.00	(Reference)	
Former	36.3	43.7	0.95	(0.82–1.09)	0.9	0.8	0.93	(0.62–1.42)	0.9253
Current	36.9	22.7	0.55	(0.47–0.64)	2.1	1.2	0.62	(0.46–0.83)	0.4750
Unknown	0.1	0.1			0.1	0.1			
**Drinking**									
Never	20.4	18.2		(Reference)	67.3	63.8	1.00	(Reference)	
Former	6.2	6.5	1.15	(0.89–1.50)	1.5	1.4	1.19	(0.87–1.62)	0.8693
Current	73.4	75.2	1.31	(1.13–1.52)	31.0	34.6	1.38	(1.27–1.50)	0.5495
Unknown	0.0	0.2			0.1	0.2			

^a^Adjusted for age, education, income, marital status, job, BMI, smoking status, and drinking status.

### Associations among regular exercise, biomarkers and risk of CMD

The associations between regular exercise at baseline and biomarkers at baseline (Supplementary Table S5), between regular exercise at baseline and the risk of CMD (Supplementary Table S6), and between biomarkers at baseline and CMD (Supplementary Table S7) were mostly consistent between men and women. Among the 42 biomarkers, regular exercise was associated with 20 biomarkers in men and 27 biomarkers in women, and 26 biomarkers in men and 29 biomarkers in women were associated with CMD. Although the associations were not significant, regular exercise seemed to protect against CMD during the follow-up.

### Mediation effects of biomarkers

**Figure 1:**
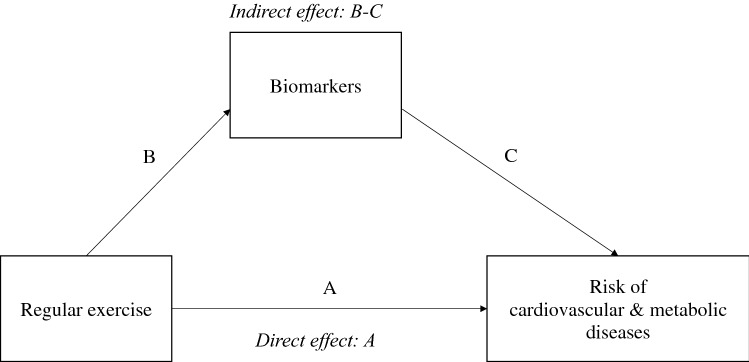
The conceptual diagram of mediation analysis. Direct effect of regular exercise on the risk of cardiovascular & metabolic conditions (CMD) is path A. Indirect effect of regular exercise on the risk of CMD mediated by each biomarker is path B and C.

We performed mediation analysis to examine the effects of biomarkers on the relationships between exercise and CMD regarding causal links (Fig. 1). Fourteen biomarkers in men and 16 biomarkers in women were shown to be the significant mediators. In particular, triglyceride showed the largest proportion explained by the indirect effect between regular exercise and the risk of CMD in both men and women, at 23.79% and 58.20%, respectively (Table 2). Waist-hip ratio, γ-glutamyl transpeptidase (γ-GTP), C-reactive protein (CRP), and white blood cell count were significant mediators in both men and women. Indirect effects of regular exercise on the risk of any CMD were observed via body composition-related markers (lean body mass, muscle mass, cell mass, protein mass, and mineral mass), hemoglobin A1c (HbA1c), albumin, alkaline phosphatase (ALP), and red blood cell count in men and via obesity-related markers (waist circumference), pulse, high-density lipoprotein (HDL), direct bilirubin, indirect bilirubin, hematocrit, and platelet count in women (Table 2).

**Table 2:** Direct and indirect effects of regular exercise on the risk of any cardiovascular & metabolic disease (no. of disease ≥ 1).

Class	Mediators	Direct effect			Indirect effect		
	Markers	HR	(95% CI)	Proportion (%)	HR	(95% CI)	Proportion (%)
**(A) Men**							
Obesity-related	Waist hip ratio	0.8949	(0.7636–1.0484)	80.53	0.9735	(0.9563–0.9883)	19.47
Body composition	Lean body mass	0.8806	(0.7518–1.0312)	92.28	0.9894	(0.9784–0.9976)	7.72
	Muscle mass	0.8812	(0.7523–1.0319)	92.40	0.9897	(0.9785–0.9980)	7.60
	Cell mass	0.8814	(0.7524–1.0321)	92.46	0.9898	(0.9785–0.9984)	7.54
	Protein mass	0.8816	(0.7526–1.0323)	92.18	0.9894	(0.9781–0.9979)	7.82
	Mineral mass	0.8772	(0.7490–1.0272)	95.27	0.9935	(0.9845–0.9997)	4.73
Lipid	Triglyceride	0.9025	(0.7706–1.0567)	76.21	0.9685	(0.9495–0.9852)	23.79
Glucose level	HbA1c	0.8922	(0.7616–1.0449)	83.91	0.9784	(0.9579–0.9977)	16.09
Liver function	Albumin	0.8642	(0.7378–1.0119)	93.64	1.0100	(1.0005–1.0221)	6.36
	ALP	0.8695	(0.7424–1.0181)	95.25	1.0070	(1.0001–1.0171)	4.75
	γ-GTP	0.8879	(0.7582–1.0395)	87.49	0.9832	(0.9676–0.9967)	12.51
Hematology	Red blood cell count	0.8839	(0.7546–1.0351)	92.94	0.9907	(0.9796–0.9993)	7.06
Inflammation	CRP	0.8889	(0.7588–1.0409)	87.78	0.9837	(0.9698–0.9950)	12.22
	White blood cell count	0.8853	(0.7554–1.0372)	90.55	0.9899	(0.9785–0.9987)	7.44
**(B) Women**							
Blood pressure	Pulse	0.9511	(0.8599–1.0517)	88.91	0.9938	(0.9874–0.9992)	11.09
Obesity-related	Waist circumference	0.9539	(0.8623–1.0549)	86.21	0.9925	(0.9854–0.9986)	13.79
	Hip circumference	0.9362	(0.8463–1.0353)	89.65	1.0076	(1.0029–1.0138)	10.35
	Waist hip ratio	0.9535	(0.8621–1.0544)	83.63	0.9907	(0.9840–0.9961)	16.37
Renal function	Uric acid	0.9427	(0.8524–1.0423)	92.27	1.0050	(1.0010–1.0102)	7.73
Lipid	HDL	0.9554	(0.8638–1.0565)	80.12	0.9887	(0.9816–0.9946)	19.88
	Triglyceride	0.9750	(0.8816–1.0780)	41.80	0.9654	(0.9525–0.9774)*	58.20
Liver function	γ-GTP	0.9576	(0.8658–1.0589)	81.80	0.9904	(0.9829–0.9969)	18.20
	Direct bilirubin	0.9531	(0.8618–1.0540)	88.95	0.9941	(0.9881–0.9991)	11.05
	Indirect bilirubin	0.9421	(0.8518–1.0419)	91.93	1.0052	(1.0005–1.0110)	8.07
Hematology	Hemoglobin	0.9396	(0.8496–1.0391)	89.24	1.0075	(1.0022–1.0140)	10.76
	Hematocrit	0.9392	(0.8492–1.0386)	88.08	1.0085	(1.0032–1.0151)	11.92
	MCV	0.9504	(0.8593–1.0509)	92.58	0.9959	(0.9912–0.9993)	7.42
	Platelet count	0.9552	(0.8636–1.0564)	84.03	0.9913	(0.9847–0.9969)	15.97
Inflammation	CRP	0.9547	(0.8632–1.0556)	80.31	0.9887	(0.9814–0.9946)	19.69
	White blood cell count	0.9506	(0.8595–1.0512)	90.47	0.9947	(0.9893–0.9989)	9.53

(A) Age as time scale and adjusted for education level, income, marital status, occupation, smoking status, drinking status, and BMI.

ALP, alkaline phosphatase; γ-GTP, γ-glutamyl transpeptidase; CRP, C-reactive protein.

(B) Age as time scale and adjusted for education level, income, marital status, occupation, smoking status, drinking status, BMI, and menopause status.

HDL, high density lipoprotein-cholesterol; γ-GTP, γ-glutamyl transpeptidase; MCV, mean corpuscular volume; CRP, C-reactive protein.

*FDR *p* value < 0.05.

### Networks of biomarkers and relationships among regular exercise, biomarkers and risk of CMD

**Figure 2:**
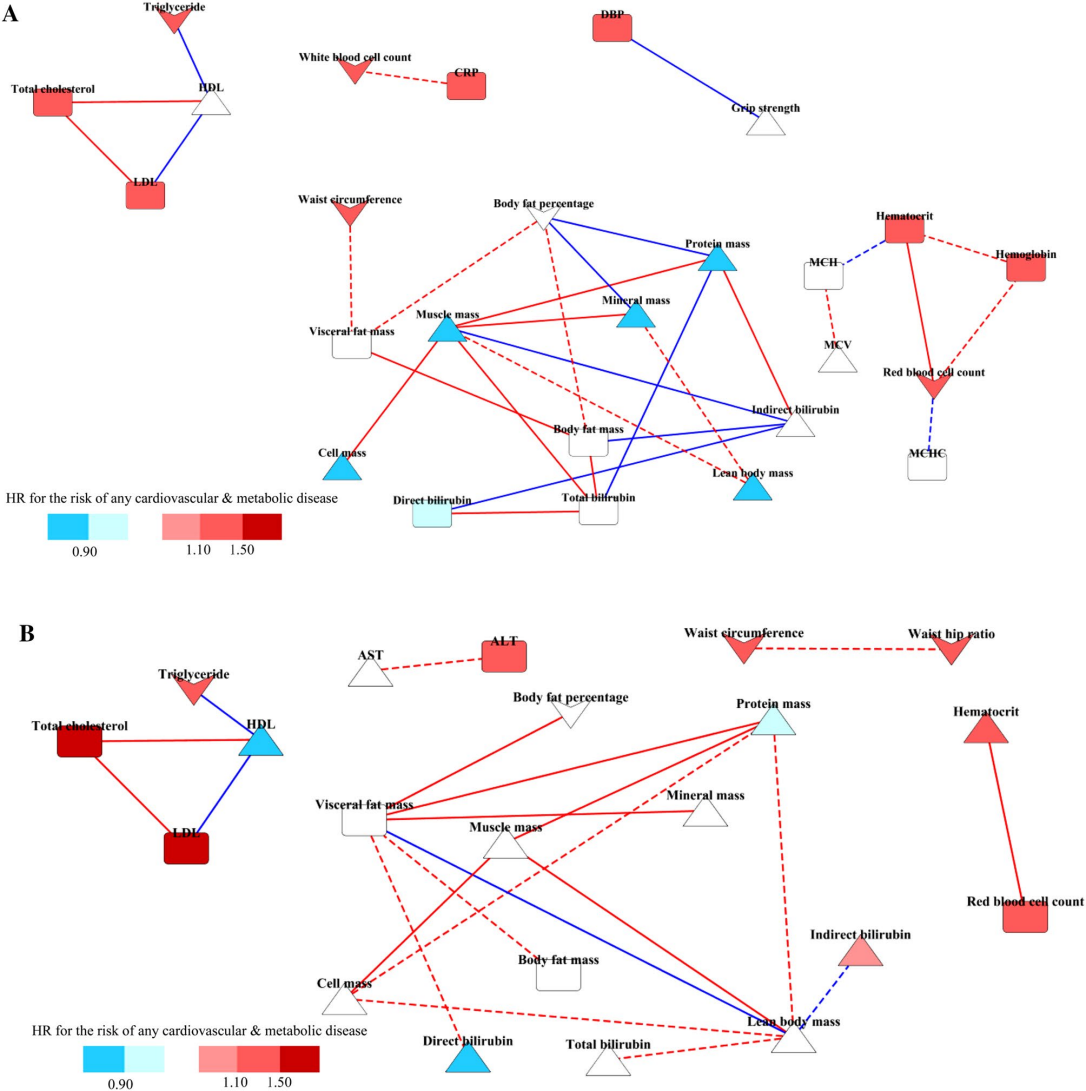
Networks of biomarkers showing their associations with regular exercise and risk of one or more diseases. Networks were constructed based on the differential correlations between partial correlation coefficients of the exercise and non-exercise groups adjusted for age. Twenty-six nodes and 31 edges in men (**A**) and 21 nodes and 21 edges in women (**B**). Δ: positive associations with regular exercise, ∀: negative associations with regular exercise, red nodes: positive associations with the risks of one or more chronic diseases, blue nodes: negative associations with the risks of one or more chronic diseases. Solid edges: higher correlations in the exercise group, dotted edges: higher correlations in the non-exercise group, red edges: positive correlations, blue edges: negative correlations. Networks were visualized by Cytoscape software (ver.3.7.2). DBP, diastolic blood pressure; HDL, high density lipoprotein-cholesterol; LDL, low density lipoprotein-cholesterol; AST, aspartate aminotransferase; ALT, alanine aminotransferase; MCV, mean corpuscular volume; MCH, mean corpuscular hemoglobin; MCHC, mean corpuscular hemoglobin concentration; CRP, C-reactive protein.

Figure 2 shows the differential correlation networks (presented by edges) constructed for men and women separately. Biomarkers are presented by nodes, and the color shows the association between biomarkers and the risk of CMD. Overall, biomarkers were clustered similarly in both men and women. Lipid markers (triglyceride, HDL, LDL, and total cholesterol) were clustered via the solid lines, which means stronger correlations in the exercise group than in the non-exercise group. Body composition-related markers and bilirubins were clustered separately. Muscle mass had the most edges that were more strongly correlated in the exercise group and was linked to body composition-related markers (cell mass, mineral mass, and protein mass) for men, whereas visceral fat mass had the most edges that were more strongly correlated in the exercise group and was linked to obesity-related markers (body fat percentage and body fat mass) for women. CRP and white blood cell count, as significant mediators between exercise and the risk of CMD in both men and women, were observed only in the network for men, and their correlation was stronger in the non-exercise group. Waist-hip ratio and waist circumference were shown only in the network for women, and their correlation was also stronger in the non-exercise group.

On the network, the nodes representing a risk for each disease suggested that obesity-related markers (waist circumference, visceral fat mass, body fat percentage, and body fat mass) were more likely to contribute to the risk of diabetes (Supplementary Fig. S1) and that markers of body composition (muscle mass, protein mass, cell mass, and lean body mass) were more likely to influence the risk of dyslipidemia (Supplementary Fig. S2). Notable markers for hypertension were not observed in the network for women, while the markers of body composition (muscle mass, protein mass, cell mass, and lean body mass) showed a protective role and diastolic blood pressure (DBP) showed a risk effect on hypertension (Supplementary Fig. S3).

## Discussion

This study examined the health benefit effects of regular exercise for preventing CMD by assessing the associations between exercise and biomarkers, the associations between biomarkers and CMD, and the mediation effects of biomarkers on the relationship between exercise and CMD. Among 42 biomarkers, we observed significant mediators for 14 of the biomarkers in men and 16 of the biomarkers in women. Especially, triglyceride showed a noteworthy mediation effect on decreasing the risk of CMD with regular exercise. The associations and correlations among regular exercise, biomarkers, and CMD were visualized by constructing networks. There were some differences in the networks between men and women.

Waist-hip ratio, triglyceride, γ-GTP, CRP, and white blood cell count were significant mediators in both men and women. The associations between exercise and these markers were consistent with those reported previously^15–20^, and waist-hip ratio, triglyceride, γ-GTP, CRP, and white blood cell count are well-known risk factors for CMD^21–25^. Among these biomarkers, only triglyceride was observed in the network of both men and women. As shown in our results, exercise decreased triglyceride and triglyceride was negatively correlated with HDL, which was enhanced by exercise. HDL was also correlated with total cholesterol and LDL. All these correlations were stronger in the exercise group than in the non-exercise group, implying that the relations between lipid markers are more highly influenced by exercise. The potential biological process would be that exercise reduces triglyceride by increasing post-heparin plasma lipoprotein lipase activity, which promotes lipoprotein-mediated hydrolysis^26^. Reducing triglyceride could impact on increasing HDL^27^. Exercise can also raise HDL by inducing liver X receptor and ATP-binding cassette transporter A-1, which influence improving the reverse cholesterol transport pathway and increasing plasma HDL formation. Consequently, increased HDL leads to reduced LDL by transporting it to the liver^28–33^.

Two of the mediators (albumin and ALP) in men and four of the mediators (hip circumference, indirect bilirubin, hemoglobin, and hematocrit) in women showed risk-mediated effects (HR > 1) despite the slight magnitudes. All these biomarkers were risk factors for CMD (Supplementary Table S7), and they showed a positive association with participating in regular exercise, although albumin and ALP were not significant (Supplementary Table S5). Elevating these biomarkers after exercise has been observed in previous studies and is probably due to adverse effects from exercise, such as damage to muscle cells or hepatic or renal stress^15,34–39^. However, the magnitude of the indirect effects via these mediators and their proportion were much less than those of the other mediators, which showed beneficial mediated effects. Further studies would be needed to understand whether the acute adverse effects are neutralized by other beneficial effects during exercise.

Differences in the networks between men and women were observed in the cluster of body composition markers. Muscle mass had the most edges in the network of men, while visceral fat mass had the most edges in women. Muscle mass was linked with protein mass, mineral mass, cell mass and lean body mass. All these biomarkers were significant mediators between exercise and the risk of CMD in men. These results suggest that muscle mass possibly plays a key role among body composition markers in terms of preventing CMD when individuals participate in regular exercise. Meanwhile, visceral fat mass seems to be a major marker among the body composition markers in women, although visceral fat mass was not a significant mediator in women. In general, there is a body compositional difference between men and women: there is more muscle mass in men and more fat mass in women^40–42^. In the previous studies, women showed higher fat oxidation and lower use of muscle glycogen than men during exercise^43–48^. Sex-based differences in motivation and patterns of participation in exercise or physical activities might be related to the different biological processes of exercise on the health benefit. Women were motivated by improvements in appearance or weight loss, while men performed exercise for enjoyment or as a challenge^49–51^. Therefore, women were likely to engage in regular walking or recreational activities, whereas men preferred strengthening exercise or competitive sports^52^. Because of the differences in preference and motivation for exercising between men and women, not only the significant mediators but also the network structure based on differential correlations between the exercise and non-exercise groups might have differed.

This study has several limitations. First, information on regular exercise and diagnosis of CMD were collected via questionnaires that might be subject to recall bias. However, the questionnaire for the diagnosis of disease was validated by the Korean Centers for Disease Control and Prevention (KCDC)^53^, and we observed that the questionnaire for regular exercise also showed acceptable validity in the ongoing work (manuscript work in progress). Second, we used a binary variable, which is participation in regular exercise or not. When we used the total time exercise per week (frequency x duration) and categorical variable (no exercise, < 150 min/week, 150–300 min/week, ≥ 300 min/week), consistent results were observed. Nevertheless, we used a binary variable (yes or no) not only for the better power of analysis but also for ease of interpretation consistent with the analysis process comparing the network of the exercise group and the non-exercise group. Third, the associations between regular exercise at baseline and the risk of each CMD were not significant. The incidence of each CMD was not sufficient because the follow-up period was relatively short. Other diseases, such as cardiovascular accidents, myocardial infarction, and cancer, had incidences of less than 2%; thus, we could not include them in the study. However, we observed a tendency of decreasing the risk of CMD and lower HRs for the risk of two or more CMD. In addition, significant indirect effects of exercise on the risk of CMD were shown through a few biomarkers. Previous studies have demonstrated that significant indirect effects can be found even though the total effect is not statistically significant and it has been suggested that this may be due to the difference in statistical power for detecting those effects^54–57^. Finally, we examined the associations between regular exercise at baseline and biomarkers at baseline, which could be seen as a cross-sectional setting. However, information on regular exercise reflected the lifestyle before enrollment in this study, while biomarkers were measured after registering in this study. Therefore, we assumed that exercise habits before enrollment influenced the biomarkers at baseline and ultimately exerted effects on CMD risk. Nevertheless, this study still has strengths. We comprehensively examined prospective associations between regular exercise, biomarkers, and risk of CMD, and significant mediators were found by mediation analysis. Their relationship was shown via networks, and the networks were based on differential correlation; therefore, the networks also imply a difference in the relationship between the biomarkers according to participation in exercise or not.

## Conclusions

The current study examined the effects of exercise on CMD by evaluating the associations between regular exercise and biomarkers, the associations between biomarkers and the risks of CMD, and, finally, the mediation effect of biomarkers on the relationships between regular exercise and CMD. Visualization of these associations in the network showed comprehensive relationships and suggested the potential biological process by which participation in regular exercise could prevent the incidence of CMD via the comprehensive benefit effects on the biomarkers. Forty-two biomarkers from anthropometric measures and laboratory tests may not be a sufficient number to show comprehensive relationships or to suggest biological processes. Further studies using metabolomics or microbiome data are needed to show more comprehensive relationships and to identify notable markers that may be key factors for explaining the health benefit of exercise on preventing chronic disease and healthy aging.

## Methods

### Study population

This study used data from the HEXA study, a large-scale genomic cohort study in Korea. The HEXA study recruited 169,722 participants aged 40 to 69 years between 2002 and 2013 from 38 general hospitals and health examination centers. Baseline data was obtained when the subjects were enrolled the study. Follow-up was conducted between 2012 and 2017, and data was obtained. The study design, data collection methods, and other details have been described previously^58,59^. Informed consents were obtained from all participants, and this study was approved by the Institutional Review Board of Seoul National University Hospital, Seoul, Korea (No. 0608-018-179). This study was performed in accordance with the Declaration of Helsinki.

The HEXA-G (Health Examinees-Gem) study was updated with additional eligibility criteria and included 139,348 participants at baseline^60^. After excluding subjects with missing information regarding regular exercise at baseline and those lost to follow-up (n = 77,197), this study included 62,151 subjects. We further excluded subjects (n = 45,098) with at least one chronic disease among cancer, cerebrovascular accident, myocardial infarction, diabetes, hypertension, and dyslipidemia at baseline; missing information on chronic diseases at baseline; and missing biomarker data to conduct analyses on subjects with complete data. Thus, this study included a total of 17,053 subjects.

### Regular exercise and biomarkers at baseline

Participation in regular exercise at baseline was investigated using an interviewer-administered questionnaire. The subjects answered yes or no to the question “Do you exercise regularly enough to sweat?”. Further queries to subjects who participated in regular exercise asked about the average frequency per week and duration. This study used a binary variable (participation in regular exercise or not) to ease of interpretation consistent with the analysis process comparing the network of the exercise group and the non-exercise group.

All available biomarkers at baseline were selected from among variables measured by clinical tests and physical examinations. Pulse (beats/minutes) was measured for 30 s or 1 min following the standard procedure. Systolic blood pressure (SBP) (mmHg), and diastolic blood pressure (DBP) (mmHg) were measured twice using a standardized mercury sphygmomanometer, with the mean of the two measurements used in the analyses. Waist circumference (cm) and hip circumference (cm) were measured to the nearest 0.1 cm. Waist-hip ratio was calculated from the measured waist circumference and hip circumference. Grip strength (kg) was measured for both hands, and the average value was used. Body fat mass (kg), percent of body fat (kg), visceral fat mass (kg), lean body mass (kg), muscle mass (kg), body cell mass (kg), protein mass (kg) and mineral mass (kg) were measured by multifrequency bioelectrical impedance analysis (MF-BIA; InBody 3.0, Biospace, Seoul, Korea). Biomarkers related to renal function (blood urea nitrogen [BUN] (mg/dL), creatinine (mg/dL), and uric acid (mg/dL)), total cholesterol (mg/dL), high-density lipoprotein cholesterol (HDL) (mg/dL), low-density lipoprotein cholesterol (LDL) (mg/dL), triglyceride (mg/dL), glucose levels (fasting blood sugar (mg/dL) and hemoglobin A1c [HbA1c] (%)), liver function (albumin (g/dL), aspartate aminotransferase [AST] (IU/L), alanine aminotransferase [ALT] (IU/L), alkaline phosphatase [ALP] (IU/L), and γ-glutamyl transpeptidase [γ-GTP] (IU/L), total bilirubin (mg/dL), direct bilirubin (mg/dL), and indirect bilirubin (mg/dL)), hematology (red blood cell (million/µL), hemoglobin (g/dL), hematocrit (%), mean corpuscular volume [MCV] (fL), mean corpuscular hemoglobin [MCH] (pg), mean corpuscular hemoglobin concentration [MCHC] (g/dL), and platelet count (thousand/µL)), inflammation (C-reactive protein [CRP] level (mg/dL) and white blood cell count (thousand/µL)), and blood levels of calcium (mg/dL) were measured using laboratory instruments such as ADVIA 1650, ADVIA 1800 (Siemens Healthineers, Deerfield, IL, USA), and VARIANT II (Bio-Rad Laboratories, Hercules, CA). Blood samples were collected after at least 8 h of fasting. This study analyzed a total of 42 biomarkers.

### Incidence of CMD

Information on diabetes, hypertension, or dyslipidemia diagnosed by a doctor during the follow-up period was self-reported by questionnaire. Subjects who reported having been diagnosed with any of these diseases were further asked when they had been diagnosed. The median follow-up period was four years from baseline. The questionnaire for the diagnosis of diseases was validated and reported by the Korean Centers for Disease Control and Prevention (KCDC)^53^. The agreement of disease history between questionnaire data from HEXA and national health insurance records showed kappa indexes of 0.93 for diabetes, 0.95 for hypertension, and 0.75 for hyperlipidemia.

Each disease was used as an outcome variable. The number of diseases was summed, and the “any CMD” variable was defined as the presence of any one of the diseases. Further analyses were performed among subjects with two or more CMD.

### Covariates

Education level, income, marital status, current job, smoking and drinking habits, and menopause status were investigated using a questionnaire. Education level was categorized as < middle school, high school, and ≥ college. Income was classified as less than 2000 thousand earned, between 2000 thousand and 4000 thousand, and ≥ 4000 thousand in Korean currency (Won). Marital status was categorized as living with a spouse or living alone. The current job was categorized into office work, manual work, unemployed or housewife, and soldier or others. Information on smoking and drinking habits was collected in terms of never, former, and current use. Body mass index (BMI) was calculated using measured weight and height (kg/m^2^).

### Statistical analysis

All analyses were performed in SAS 9.4 and R software (ver. 4.0.0). Biomarkers were normal score transformed using the “gstat” package in R to make normal distributions and unify scales^61^. The standardized differences between included (n = 17,053) and excluded (n = 45,098) datasets were calculated using the “stddiff” package in R. Standardized differences greater than 0.2 were considered indicative of an imbalance between datasets^62^. Correlation coefficients of age were estimated for all potential biomarkers. Wilcox rank-sum and chi-square tests were performed to evaluate the differences in basic characteristics and biomarkers between men and women. These summary statistics and odds ratios (ORs) with 95% confidence intervals (95% CIs) from logistic regression were estimated in SAS 9.4. Age, education level, income, marital status, current job, smoking and drinking habit, and BMI at baseline were included as covariates in the statistical models. Menopause status at baseline was additionally included in the models for women. General linear models were used to examine the associations between regular exercise at baseline and biomarkers at baseline after adjusting for covariates and multiple corrections in R software. Cox proportional hazard regression models were used to examine the associations 1) between regular exercise at baseline and risks of CMD at follow-up and 2) between biomarkers at baseline and risks of CMD at follow-up adjusting covariates. Hazard ratios (HRs) with 95% CIs were estimated using the “survival” package in R software.

Mediation analysis based on Cox proportional hazard regression models with the same covariates as above was performed to examine whether regular exercise influenced the risk of CMD directly without any mediator effect or indirectly through biomarkers as the mediators (Fig. 2). When the 95% CI of the estimated indirect effect did not include 0, the indirect effects were considered statistically significant. The proportions explained by the indirect effect of regular exercise through each biomarker on the risk of CMD were calculated as the indirect effect divided by the total effect (direct effect + indirect effect). The R code used for mediation analysis has been described previously^63^.

Biomarker networks were constructed for men and women separately to comprehensively visualize the associations between regular exercise and biomarkers, relationships among biomarkers, and the effects of biomarkers on the risks of CMD. Partial correlation matrixes adjusted for age were calculated in the exercise and non-exercise groups using the “pcor” package in R. The “DiffCorr” package was used to identify significant differential correlations between the exercise and non-exercise groups. Among significant differential correlations (*p* < 0.05), partial correlations coefficients with absolute values greater than 0.1 were selected and visualized as networks in Cytoscape software (ver. 3.7.2). Correlations among biomarkers were presented as the edges. Solid edges indicated significantly larger correlations of the partial correlation coefficients in the exercise group than those in the non-exercise group. Dotted edges indicated correlations with significantly larger partial correlation coefficients in the non-exercise group than those in the exercise group. The associations between regular exercise and biomarkers were indicated by triangle direction (Δ: positive associations, ∀: negative associations). The associations between biomarkers and risks of CMD were indicated by node color (red: positive associations, blue: negative associations).

### Ethics approval and consent to participate

All participants signed consent forms, and this study was approved by the Institutional Review Board of Seoul National University Hospital, Seoul, Korea (No. 0608-018-179).

## Supplementary Information

Supplementary Information.

## Data Availability

The data underlying this study are the Health Examinee cohort, a part of the Korean Genome and Epidemiology Study (KoGES). Researchers who want to conduct studies using this data can apply for data access by submitting application form with documents such as research plan and the Institutional Review Board approval form. The relevant data requesting process and contact information in detail can be found in the following link: http://www.nih.go.kr/contents.es?mid=a50401010400#menu4_1_2.

## Abbreviations

ALP: Alkaline phosphatase
ALT: Alanine aminotransferase
AST: Aspartate aminotransferase
BMI: Body mass index
BUN: Blood urea nitrogen
CMD: Cardiovascular & metabolic diseases
CI: Confidence intervals
CRP: C-reactive protein
DBP: Diastolic blood pressure
γ-GTP: γ-Glutamyl transpeptidase
HbA1c: Hemoglobin A1c
HEXA: Health Examinees
HEXA-G: Health Examinees-Gem
HDL: High-density lipoprotein cholesterol
HR: Hazard ratios
KCDC: Korean Centers for Disease Control and Prevention
LDL: Low-density lipoprotein cholesterol
MCH: Mean corpuscular hemoglobin
MCHC: Mean corpuscular hemoglobin concentration
MCV: Mean corpuscular volume
OR: Odds ratios
SBP: Systolic blood pressure
