# Supplementary material for: Network of biomarkers and their mediation effects on the associations between regular exercise and the incidence of cardiovascular & metabolic diseases
Source: Sci Rep. 2021 Jun 17;11:12802. doi: 10.1038/s41598-021-92312-x (PMC8211674; doi:10.1038/s41598-021-92312-x)
Supplement: Supplementary file 1 — Supplementary Information. [file 41598_2021_92312_MOESM1_ESM.docx]

**Supplementary material**

Network of biomarkers and their mediation effects on the associations between regular exercise and the incidence of cardiovascular & metabolic diseases

JooYong Park^1^, Jaesung Choi^2^, Ji-Eun Kim^1^, Miyoung Lee^3^, Aesun Shin^4,5^,
Jong-koo Lee^6,7^, Daehee Kang^1,4,5^, and Ji-Yeob Choi^1,2*^

^1^Department of Biomedical Sciences, Seoul National University Graduate School, Seoul, Korea

^2^Institute of Health Policy and Management, Seoul National University Medical Research Center, Seoul, Korea

^3^College of Physical Education and Sport Science, Kookmin University, Seoul, Korea

^4^Department of Preventive Medicine, Seoul National University College of Medicine, Seoul, Korea

^5^Cancer Research Institute, Seoul National University, Seoul, Korea

^6^JW Lee Center for Global Medicine, Seoul National University College of Medicine, Seoul, Korea

^7^Department of Family Medicine, Seoul National University College of Medicine, Seoul, Korea

Supplementary Figure S1. Networks of biomarkers showing the associations with regular exercise and the risk of diabetes

Supplementary Figure S2. Networks of biomarkers showing associations between regular exercise and risk of dyslipidemia

Supplementary Figure S3. Networks of biomarkers showing the associations with regular exercise and risk of hypertension

Supplementary Table S1. Comparisons between the included and excluded datasets

Supplementary Table S2. Correlations between biomarkers and age at baseline

Supplementary Table S3. Characteristics of the study population at baseline by sex

Supplementary Table S4. Distributions of biomarkers at baseline by sex

Supplementary Table S5. Associations between participation in regular exercise and biomarkers at baseline

Supplementary Table S6. Associations between participation in regular exercise and risk of each cardiovascular & metabolic disease

Supplementary Table S7. Associations between biomarkers and the risk of each cardiovascular & metabolic disease

A


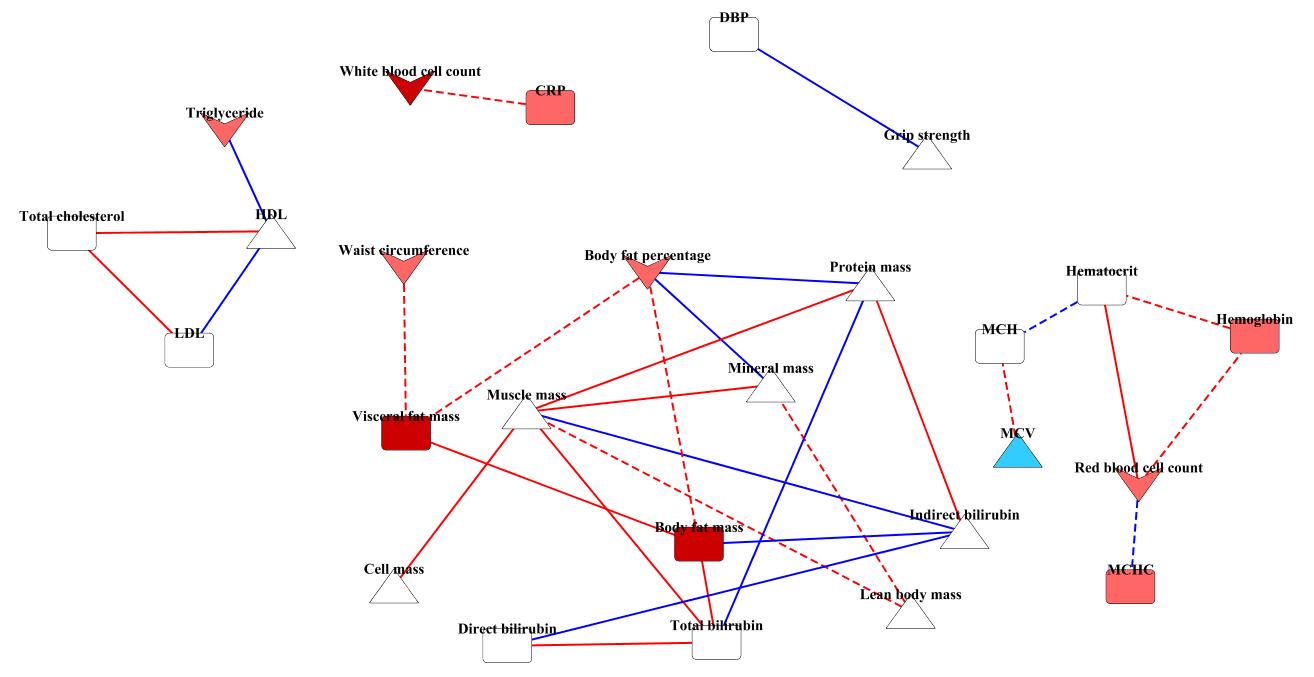


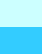

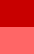


0.90

1.50


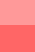


1.10

HR for diabetes

B


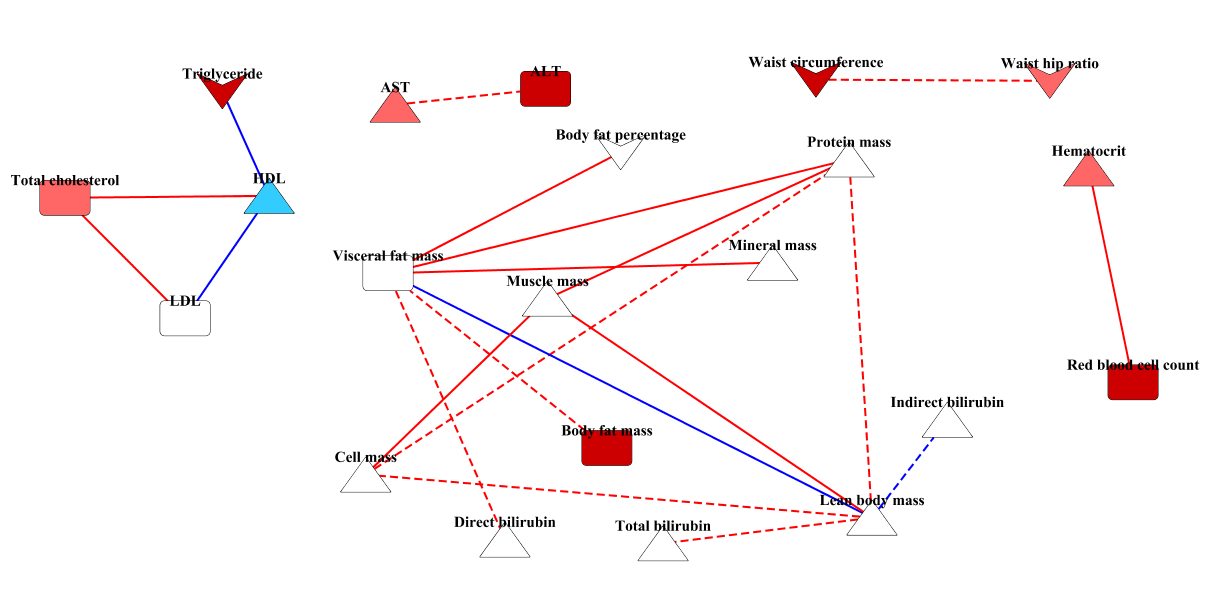


HR for diabetes


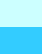

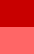


0.90

1.50


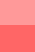


1.10

Supplementary Figure S1. Networks of biomarkers showing the associations with regular exercise and the risk of diabetes

Networks were constructed based on the differential correlations between the partial correlation coefficients of the exercise and non-exercise groups adjusted for age. Twenty-six nodes and 31 edges in men (A) and 21 nodes and 21 edges in women (B). △: positive associations with regular exercise, ∀: negative associations with regular exercise, red nodes: positive associations with risks of one or more chronic diseases, blue nodes: negative associations with risks of one or more chronic diseases. Solid edges: higher correlations in the exercise group, dotted edges: higher correlations in the non-exercise group, red edges: positive correlations, blue edges: negative correlations

Networks were visualized by Cytoscape software (ver.3.7.2).

DBP: diastolic blood pressure, HDL: high density lipoprotein-cholesterol, LDL: low density lipoprotein-cholesterol, AST: aspartate aminotransferase, ALT: alanine aminotransferase, MCV: mean corpuscular volume, MCH: mean corpuscular hemoglobin, MCHC: mean corpuscular hemoglobin concentration, CRP: C-reactive protein

A


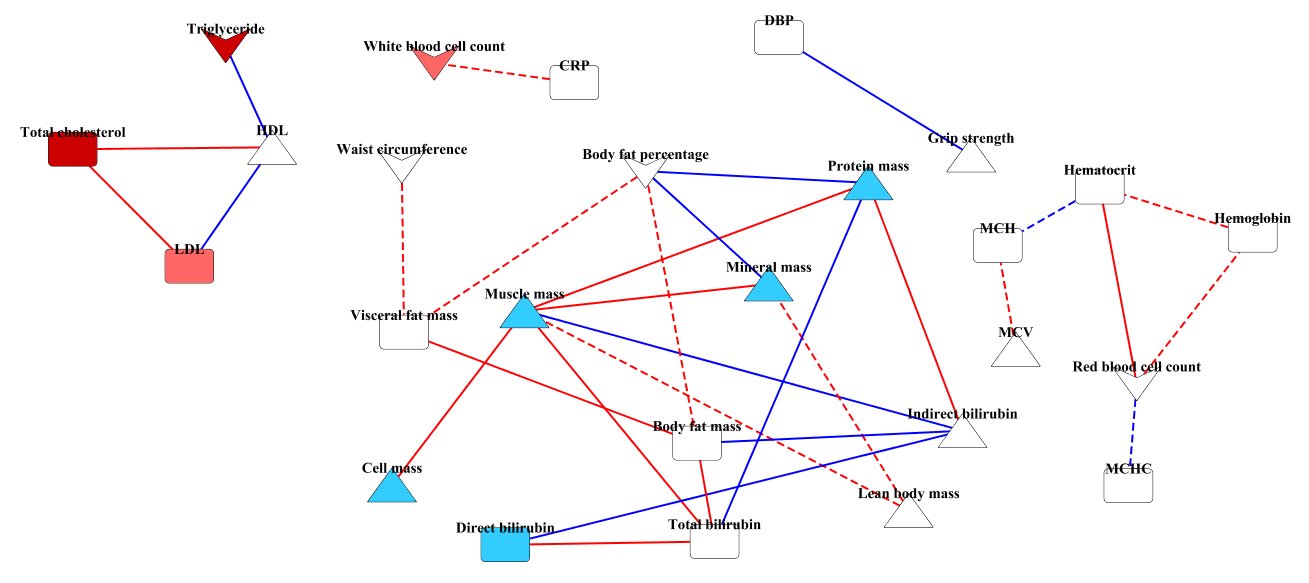


HR for dyslipidemia


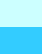

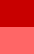


0.90

1.50


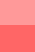


1.10

B


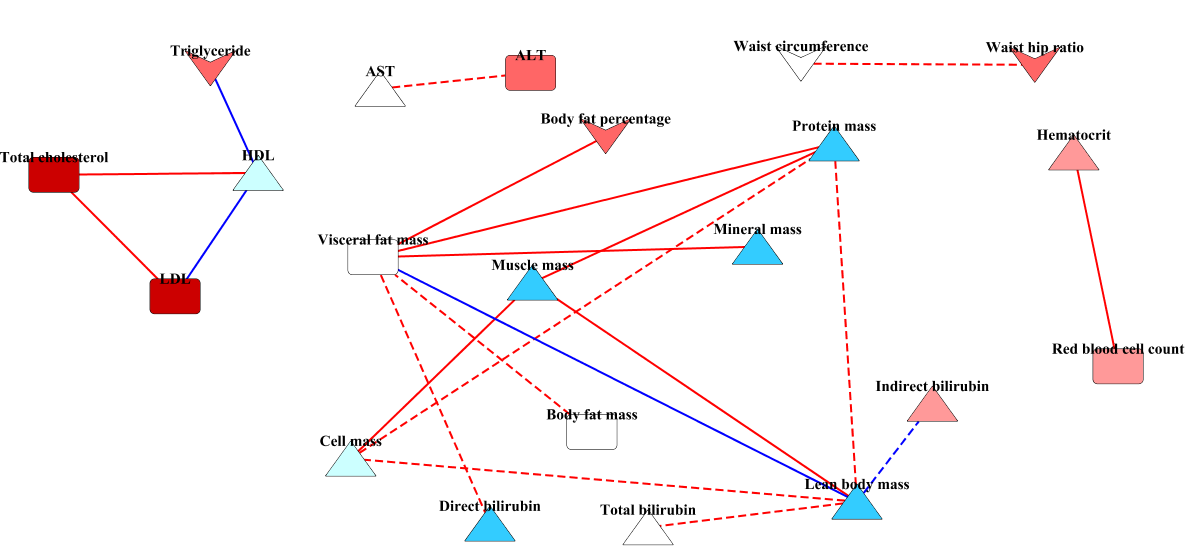


HR for dyslipidemia


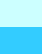

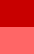


0.90

1.50


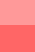


1.10

Supplementary Figure S2. Networks of biomarkers showing associations between regular exercise and risk of dyslipidemia

Networks were constructed based on the differential correlations between the partial correlation coefficients of the exercise and non-exercise groups adjusted for age. Twenty-six nodes and 31 edges in men (A) and 21 nodes and 21 edges in women (B) are shown. △: positive associations with regular exercise, ∀: negative associations with regular exercise, red nodes: positive associations with the risks of one or more chronic diseases, blue nodes: negative associations with the risks of one or more chronic diseases. Solid edges: higher correlations in the exercise group; dotted edges: higher correlations in the non-exercise group, red edges: positive correlations, blue edges: negative correlations

Networks were visualized by Cytoscape software (ver.3.7.2).

DBP: diastolic blood pressure, HDL: high density lipoprotein-cholesterol, LDL: low density lipoprotein-cholesterol, AST: aspartate aminotransferase, ALT: alanine aminotransferase, MCV: mean corpuscular volume, MCH: mean corpuscular hemoglobin, MCHC: mean corpuscular hemoglobin concentration, CRP: C-reactive protein

A


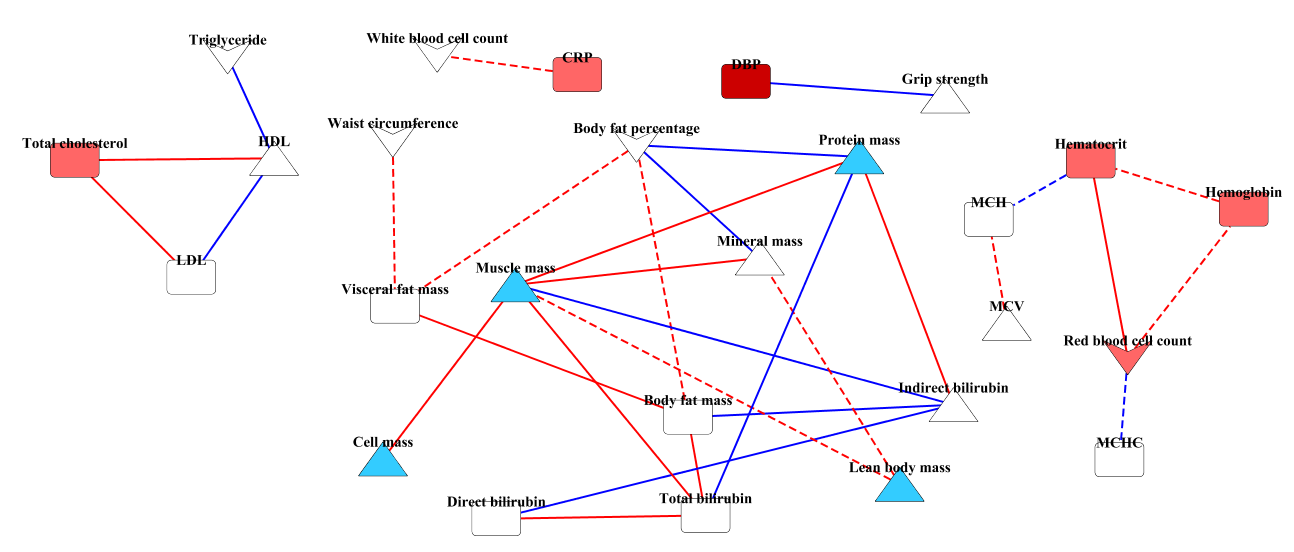


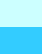

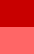


0.90

1.50


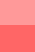


1.10

HR for hypertension

B


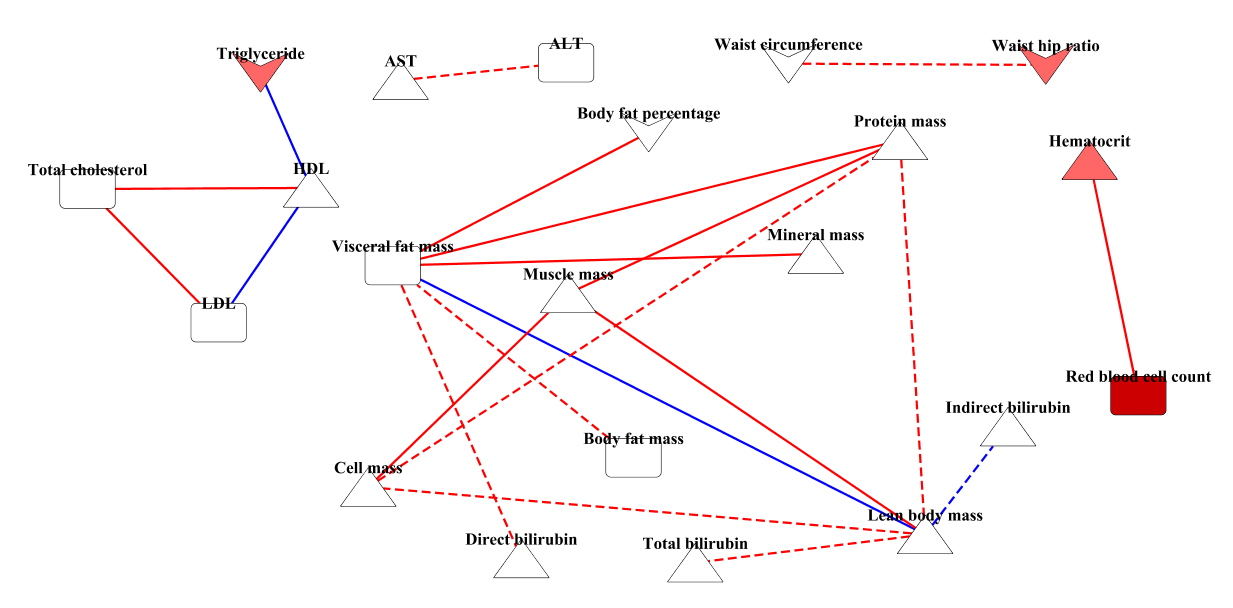


HR for hypertension


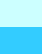

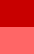


0.90

1.50


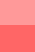


1.10

Supplementary Figure S3. Networks of biomarkers showing the associations with regular exercise and risk of hypertension

Networks were constructed based on the differential correlations between the partial correlation coefficients of the exercise and non-exercise groups adjusted for age. Twenty-six nodes and 31 edges in men (A) and 21 nodes and 21 edges in women (B). △: positive associations with regular exercise, ∀: negative associations with regular exercise, red nodes: positive associations with risks of one or more chronic diseases, blue nodes: negative associations with risks of one or more chronic diseases. Solid edges: higher correlations in the exercise group, dotted edges: higher correlations in the non-exercise group, red edges: positive correlations, blue edges: negative correlations

Networks were visualized by Cytoscape software (ver.3.7.2).

DBP: diastolic blood pressure, HDL: high density lipoprotein-cholesterol, LDL: low density lipoprotein-cholesterol, AST: aspartate aminotransferase, ALT: alanine aminotransferase, MCV: mean corpuscular volume, MCH: mean corpuscular hemoglobin, MCHC: mean corpuscular hemoglobin concentration, CRP: C-reactive protein

Supplementary Table S1. Comparisons between the included and excluded datasets

|  | Included dataset | | Excluded dataset | |  |
| --- | --- | --- | --- | --- | --- |
|  | N=17,053 | | N=45,098 | |  |
|  | N | (%) | N | (%) | Standardized difference^*^ |
| Sex |  | |  | |  |
| Men | 5,390 | (31.6) | 15,471 | (34.3) |  |
| Women | 11,663 | (68.4) | 29,627 | (65.7) |  |
| Age, mean ± SD (years) | 52.6 ± 7.75 | | 53.8 ± 7.81 | | 0.151 |
| 40–44 | 3,213 | (18.8) | 6,508 | (14.4) | 0.166 |
| 45–49 | 2,833 | (16.6) | 7,718 | (17.1) |  |
| 50­54 | 4,110 | (24.1) | 10,174 | (22.6) |  |
| 55–59 | 3,283 | (19.3) | 8,638 | (19.2) |  |
| 60–64 | 2,369 | (13.9) | 7,428 | (16.5) |  |
| 65–69 | 1,245 | (7.3) | 4,632 | (10.3) |  |
| Education |  |  |  |  |  |
| ≤Middle school | 4,730 | (27.7) | 14,516 | (32.2) | 0.107 |
| High school | 7,551 | (44.3) | 18,965 | (42.1) |  |
| ≥College | 4,719 | (27.7) | 11,107 | (24.6) |  |
| Unknown | 53 | (0.3) | 510 | (1.1) |  |
| Income (₩10,000) |  |  |  |  |  |
| <200 | 4,073 | (23.9) | 12,986 | (28.8) | 0.217 |
| 200–400 | 7,475 | (43.8) | 17,270 | (38.3) |  |
| ≥400 | 5,130 | (30.1) | 9,156 | (20.3) |  |
| Unknown | 375 | (2.2) | 5,686 | (12.6) |  |
| Marital status |  |  |  |  |  |
| Living with spouse | 15,416 | (90.4) | 40,824 | (90.5) | 0.010 |
| Living alone | 1,620 | (9.5) | 4,152 | (9.2) |  |
| Unknown | 17 | (0.1) | 122 | (0.3) |  |
| Current occupation |  |  |  |  |  |
| Office | 3,859 | (22.6) | 8,228 | (18.2) | 0.119 |
| Manual | 5,545 | (32.5) | 13,745 | (30.5) |  |
| Unemployed/housewife | 7,488 | (43.9) | 21,627 | (48.0) |  |
| Soldier/etc. | 112 | (0.7) | 253 | (0.6) |  |
| Unknown | 49 | (0.3) | 1,245 | (2.8) |  |
| BMI, kg/m^2^ |  |  |  |  |  |
| <18.5 | 354 | (2.1) | 679 | (1.5) | 0.094 |
| 18.5–23 | 6,929 | (40.6) | 16,713 | (37.1) |  |
| 23–25 | 4,705 | (27.6) | 12,855 | (28.5) |  |
| 25–30 | 4,669 | (27.4) | 13,551 | (30.1) |  |
| ≥30 | 396 | (2.3) | 1,276 | (2.8) |  |
| Unknown | 0 | (0.0) | 24 | (0.1) |  |
| Smoking |  |  |  |  |  |
| Never | 13,026 | (76.4) | 33,311 | (73.9) | 0.059 |
| Former | 2,294 | (13.5) | 6,927 | (15.4) |  |
| Current | 1,720 | (10.1) | 4,660 | (10.3) |  |
| Unknown | 13 | (0.1) | 200 | (0.4) |  |
| Drinking |  |  |  |  |  |
| Never | 8,662 | (50.8) | 23,871 | (52.9) | 0.083 |
| Former | 512 | (3.0) | 1,839 | (4.1) |  |
| Current | 7,855 | (46.1) | 19,224 | (42.6) |  |
| Unknown | 24 | (0.1) | 164 | (0.4) |  |
|  |  |  |  |  |  |
| Regular exercise, mean ± SD | 180.1 ± 255.6 | | 170.7 ± 238.4 | | 0.038 |
| No | 7,585 | (44.5) | 20,759 | (46.0) | 0.031 |
| Yes | 9,468 | (55.5) | 24,339 | (54.0) |  |

^*^ Standardized difference between included dataset and excluded dataset without missing value

Supplementary Table S2. Correlations between biomarkers and age at baseline

(A) Men

|  |  |  |  |  |  | Normal score transformation | | | |
| --- | --- | --- | --- | --- | --- | --- | --- | --- | --- |
|  | Pearson correlation | p (Pearson) | Spearman  rank-order correlation | p (Spearman) |  | Pearson correlation | p (Pearson) | Spearman  rank-order correlation | p (Spearman) |
| Pulse | -0.0776 | <.0001 | -0.0870 | <.0001 |  | -0.0819 | <.0001 | -0.0870 | <.0001 |
| SBP | 0.0718 | <.0001 | 0.0789 | <.0001 |  | 0.0731 | <.0001 | 0.0789 | <.0001 |
| DBP | -0.0351 | 0.0099 | -0.0334 | 0.0141 |  | -0.0330 | 0.0154 | -0.0339 | 0.0129 |
| Waist circumference | 0.0419 | 0.0021 | 0.0483 | 0.0004 |  | 0.0422 | 0.002 | 0.0480 | 0.0004 |
| Hip circumference | -0.1000 | <.0001 | -0.1065 | <.0001 |  | -0.1044 | <.0001 | -0.1067 | <.0001 |
| Waist hip ratio | 0.1645 | <.0001 | 0.1731 | <.0001 |  | 0.1697 | <.0001 | 0.1723 | <.0001 |
| Body fat mass | -0.0925 | <.0001 | -0.0800 | <.0001 |  | -0.0860 | <.0001 | -0.0800 | <.0001 |
| Body fat percentage | 0.0200 | 0.1421 | 0.0172 | 0.2068 |  | 0.0164 | 0.2284 | 0.0171 | 0.2095 |
| Visceral fat mass | -0.2779 | <.0001 | -0.2751 | <.0001 |  | -0.2809 | <.0001 | -0.2751 | <.0001 |
| Lean body mass | -0.2804 | <.0001 | -0.2789 | <.0001 |  | -0.2842 | <.0001 | -0.2790 | <.0001 |
| Muscle mass | -0.3131 | <.0001 | -0.3122 | <.0001 |  | -0.3165 | <.0001 | -0.3123 | <.0001 |
| Cell mass | -0.2876 | <.0001 | -0.2926 | <.0001 |  | -0.2960 | <.0001 | -0.2928 | <.0001 |
| Protein mass | -0.2208 | <.0001 | -0.2079 | <.0001 |  | -0.2193 | <.0001 | -0.2081 | <.0001 |
| Mineral mass | -0.0472 | 0.0005 | -0.0333 | 0.0146 |  | -0.0371 | 0.0064 | -0.0337 | 0.0134 |
| BUN | 0.2065 | <.0001 | 0.2063 | <.0001 |  | 0.2085 | <.0001 | 0.2045 | <.0001 |
| Creatinine | 0.0336 | 0.0136 | 0.0229 | 0.0931 |  | 0.0286 | 0.0359 | 0.0222 | 0.1034 |
| Uric acid | -0.0256 | 0.0602 | -0.0343 | 0.0118 |  | -0.0304 | 0.0256 | -0.0346 | 0.0112 |
| Total cholesterol | -0.0523 | 0.0001 | -0.0491 | 0.0003 |  | -0.0523 | 0.0001 | -0.0493 | 0.0003 |
| HDL | 0.0285 | 0.0368 | 0.0215 | 0.1139 |  | 0.0206 | 0.131 | 0.0215 | 0.1142 |
| LDL | 0.0020 | 0.8821 | 0.0034 | 0.8037 |  | 0.0013 | 0.9226 | 0.0034 | 0.8055 |
| Triglyceride | -0.1042 | <.0001 | -0.1131 | <.0001 |  | -0.1098 | <.0001 | -0.1132 | <.0001 |
| Fasting blood sugar | 0.0663 | <.0001 | 0.0862 | <.0001 |  | 0.0826 | <.0001 | 0.0858 | <.0001 |
| HbA1c | 0.1259 | <.0001 | 0.1623 | <.0001 |  | 0.1581 | <.0001 | 0.1615 | <.0001 |
| Albumin | -0.2081 | <.0001 | -0.2100 | <.0001 |  | -0.2102 | <.0001 | -0.2095 | <.0001 |
| AST | -0.0023 | 0.8691 | 0.0148 | 0.2782 |  | 0.0138 | 0.3101 | 0.0144 | 0.2913 |
| ALT | -0.1349 | <.0001 | -0.1521 | <.0001 |  | -0.1501 | <.0001 | -0.1523 | <.0001 |
| ALP | -0.0020 | 0.8839 | 0.0121 | 0.3762 |  | 0.0053 | 0.6994 | 0.0120 | 0.3804 |
| γ-GTP | -0.0648 | <.0001 | -0.1049 | <.0001 |  | -0.0990 | <.0001 | -0.1050 | <.0001 |
| Total bilirubin | -0.0326 | 0.0168 | -0.0266 | 0.0506 |  | -0.0252 | 0.0641 | -0.0282 | 0.0385 |
| Direct bilirubin | -0.0287 | 0.0349 | -0.0214 | 0.1168 |  | -0.0266 | 0.0507 | -0.0250 | 0.0670 |
| Indirect bilirubin | -0.0299 | 0.0280 | -0.0262 | 0.0547 |  | -0.0228 | 0.0941 | -0.0283 | 0.0379 |
| Red blood cell count | -0.2399 | <.0001 | -0.2438 | <.0001 |  | -0.2431 | <.0001 | -0.2439 | <.0001 |
| Hemoglobin | -0.1719 | <.0001 | -0.1750 | <.0001 |  | -0.1752 | <.0001 | -0.1750 | <.0001 |
| Hematocrit | -0.1620 | <.0001 | -0.1619 | <.0001 |  | -0.1627 | <.0001 | -0.1620 | <.0001 |
| MCV | 0.1624 | <.0001 | 0.1773 | <.0001 |  | 0.1727 | <.0001 | 0.1772 | <.0001 |
| MCH | 0.1146 | <.0001 | 0.1369 | <.0001 |  | 0.1311 | <.0001 | 0.1367 | <.0001 |
| MCHC | -0.0655 | <.0001 | -0.0692 | <.0001 |  | -0.0695 | <.0001 | -0.0690 | <.0001 |
| Platelet count | -0.1054 | <.0001 | -0.1160 | <.0001 |  | -0.1089 | <.0001 | -0.1161 | <.0001 |
| CRP | 0.0443 | 0.0011 | 0.0393 | 0.0039 |  | 0.0359 | 0.0084 | 0.0391 | 0.0041 |
| White blood cell count | -0.0538 | <.0001 | -0.0494 | 0.0003 |  | -0.0535 | <.0001 | -0.0494 | 0.0003 |
| Grip strength | -0.2831 | <.0001 | -0.3219 | <.0001 |  | -0.3076 | <.0001 | -0.3219 | <.0001 |
| Calcium level | -0.1085 | <.0001 | -0.1097 | <.0001 |  | -0.1104 | <.0001 | -0.1095 | <.0001 |

SBP: systolic blood pressure, DBP: diastolic blood pressure, BUN: blood urea nitrogen, HDL: high density lipoprotein-cholesterol, LDL: low density lipoprotein-cholesterol, HbA1c: hemoglobin A1c, AST: aspartate aminotransferase, ALT: alanine aminotransferase, ALP: alkaline phosphatase, γ-GTP: γ-glutamyl transpeptidase, MCV: mean corpuscular volume, MCH: mean corpuscular hemoglobin, MCHC: mean corpuscular hemoglobin concentration, CRP: C-reactive protein

(B) Women

|  |  |  |  |  |  | Normal score transformation | | | |
| --- | --- | --- | --- | --- | --- | --- | --- | --- | --- |
|  | Pearson correlation | p (Pearson) | Spearman  rank-order correlation | p (Spearman) |  | Pearson correlation | p (Pearson) | Spearman  rank-order correlation | p (Spearman) |
| Pulse | -0.0748 | <.0001 | -0.0834 | <.0001 |  | -0.0761 | <.0001 | -0.0828 | <.0001 |
| SBP | 0.2356 | <.0001 | 0.2460 | <.0001 |  | 0.2395 | <.0001 | 0.2464 | <.0001 |
| DBP | 0.1588 | <.0001 | 0.1681 | <.0001 |  | 0.1628 | <.0001 | 0.1691 | <.0001 |
| Waist circumference | 0.2196 | <.0001 | 0.2266 | <.0001 |  | 0.2243 | <.0001 | 0.2268 | <.0001 |
| Hip circumference | 0.0248 | 0.0075 | 0.0190 | 0.0402 |  | 0.0243 | 0.0086 | 0.0192 | 0.0384 |
| Waist hip ratio | 0.2826 | <.0001 | 0.2845 | <.0001 |  | 0.2851 | <.0001 | 0.2847 | <.0001 |
| Body fat mass | 0.1397 | <.0001 | 0.1618 | <.0001 |  | 0.1539 | <.0001 | 0.1619 | <.0001 |
| Body fat percentage | 0.2713 | <.0001 | 0.2747 | <.0001 |  | 0.2720 | <.0001 | 0.2747 | <.0001 |
| Visceral fat mass | -0.1745 | <.0001 | -0.1695 | <.0001 |  | -0.1767 | <.0001 | -0.1694 | <.0001 |
| Lean body mass | -0.1900 | <.0001 | -0.1854 | <.0001 |  | -0.1921 | <.0001 | -0.1852 | <.0001 |
| Muscle mass | -0.2241 | <.0001 | -0.2214 | <.0001 |  | -0.2285 | <.0001 | -0.2212 | <.0001 |
| Cell mass | -0.2474 | <.0001 | -0.2484 | <.0001 |  | -0.2523 | <.0001 | -0.2476 | <.0001 |
| Protein mass | -0.0227 | 0.0143 | -0.0064 | 0.487 |  | -0.0156 | 0.0911 | -0.0057 | 0.539 |
| Mineral mass | 0.1952 | <.0001 | 0.2274 | <.0001 |  | 0.2213 | <.0001 | 0.2274 | <.0001 |
| BUN | 0.3119 | <.0001 | 0.3321 | <.0001 |  | 0.3270 | <.0001 | 0.3320 | <.0001 |
| Creatinine | 0.0420 | <.0001 | 0.0501 | <.0001 |  | 0.0561 | <.0001 | 0.0501 | <.0001 |
| Uric acid | 0.1559 | <.0001 | 0.1602 | <.0001 |  | 0.1559 | <.0001 | 0.1608 | <.0001 |
| Total cholesterol | 0.2182 | <.0001 | 0.2335 | <.0001 |  | 0.2216 | <.0001 | 0.2337 | <.0001 |
| HDL | -0.1046 | <.0001 | -0.1072 | <.0001 |  | -0.1087 | <.0001 | -0.1067 | <.0001 |
| LDL | 0.2206 | <.0001 | 0.2355 | <.0001 |  | 0.2221 | <.0001 | 0.2355 | <.0001 |
| Triglyceride | 0.1438 | <.0001 | 0.2067 | <.0001 |  | 0.1977 | <.0001 | 0.2068 | <.0001 |
| Fasting blood sugar | 0.1298 | <.0001 | 0.1773 | <.0001 |  | 0.1707 | <.0001 | 0.1776 | <.0001 |
| HbA1c | 0.2440 | <.0001 | 0.3217 | <.0001 |  | 0.3134 | <.0001 | 0.3215 | <.0001 |
| Albumin | -0.0253 | 0.0062 | -0.0112 | 0.2247 |  | -0.0230 | 0.0132 | -0.0100 | 0.2813 |
| AST | 0.1701 | <.0001 | 0.3168 | <.0001 |  | 0.3026 | <.0001 | 0.3171 | <.0001 |
| ALT | 0.1143 | <.0001 | 0.2514 | <.0001 |  | 0.2380 | <.0001 | 0.2517 | <.0001 |
| ALP | 0.2795 | <.0001 | 0.3265 | <.0001 |  | 0.3039 | <.0001 | 0.3265 | <.0001 |
| γ-GTP | 0.0823 | <.0001 | 0.1942 | <.0001 |  | 0.1850 | <.0001 | 0.1943 | <.0001 |
| Total bilirubin | -0.0477 | <.0001 | -0.0417 | <.0001 |  | -0.0389 | <.0001 | -0.0403 | <.0001 |
| Direct bilirubin | -0.1004 | <.0001 | -0.0969 | <.0001 |  | -0.0834 | <.0001 | -0.0829 | <.0001 |
| Indirect bilirubin | -0.0171 | 0.0645 | -0.0118 | 0.2014 |  | -0.0086 | 0.3548 | -0.0102 | 0.2705 |
| Red blood cell count | -0.0266 | 0.0041 | -0.0223 | 0.0162 |  | -0.0268 | 0.0038 | -0.0221 | 0.017 |
| Hemoglobin | 0.1350 | <.0001 | 0.1043 | <.0001 |  | 0.1142 | <.0001 | 0.1047 | <.0001 |
| Hematocrit | 0.1358 | <.0001 | 0.1179 | <.0001 |  | 0.1270 | <.0001 | 0.1180 | <.0001 |
| MCV | 0.2092 | <.0001 | 0.1860 | <.0001 |  | 0.1960 | <.0001 | 0.1861 | <.0001 |
| MCH | 0.1895 | <.0001 | 0.1458 | <.0001 |  | 0.1580 | <.0001 | 0.1461 | <.0001 |
| MCHC | 0.0706 | <.0001 | 0.0278 | 0.0027 |  | 0.0416 | <.0001 | 0.0280 | 0.0025 |
| Platelet count | -0.0920 | <.0001 | -0.0886 | <.0001 |  | -0.0901 | <.0001 | -0.0885 | <.0001 |
| CRP | 0.0619 | <.0001 | 0.1922 | <.0001 |  | 0.1876 | <.0001 | 0.1924 | <.0001 |
| White blood cell count | -0.0330 | 0.0004 | -0.0498 | <.0001 |  | -0.0357 | 0.0001 | -0.0498 | <.0001 |
| Grip strength | -0.2556 | <.0001 | -0.2855 | <.0001 |  | -0.2815 | <.0001 | -0.2854 | <.0001 |
| Calcium level | 0.1684 | <.0001 | 0.1942 | <.0001 |  | 0.1731 | <.0001 | 0.1946 | <.0001 |

SBP: systolic blood pressure, DBP: diastolic blood pressure, BUN: blood urea nitrogen, HDL: high density lipoprotein-cholesterol, LDL: low density lipoprotein-cholesterol, HbA1c: hemoglobin A1c, AST: aspartate aminotransferase, ALT: alanine aminotransferase, ALP: alkaline phosphatase, γ-GTP: γ-glutamyl transpeptidase, MCV: mean corpuscular volume, MCH: mean corpuscular hemoglobin, MCHC: mean corpuscular hemoglobin concentration, CRP: C-reactive protein

Supplementary Table S3. Characteristics of the study population at baseline by sex

|  | Men | | Women | |
| --- | --- | --- | --- | --- |
|  | N=5,390 | | N=11,663 | |
|  | N | (%) | N | (%) |
| **Age,** Mean ± SD (years) | 54.0 ± 8.22 | | 51.9 ± 7.43 | |
| 40–44 | 935 | (17.4) | 2,278 | (19.5) |
| 45–49 | 688 | (12.8) | 2,145 | (18.4) |
| 50–54 | 1,076 | (20.0) | 3,034 | (26.0) |
| 55–59 | 1,109 | (20.6) | 2,174 | (18.6) |
| 60–64 | 969 | (18.0) | 1,400 | (12.0) |
| 65–69 | 613 | (11.4) | 632 | (5.4) |
| **Education** |  |  |  |  |
| ≤Middle school | 1,044 | (19.4) | 3,686 | (31.6) |
| High school | 2,149 | (39.9) | 5,402 | (46.3) |
| ≥College | 2,178 | (40.4) | 2,541 | (21.8) |
| Unknown | 19 | (0.4) | 34 | (0.3) |
| **Income (₩10,000)** |  |  |  |  |
| <200 | 1,149 | (21.3) | 2,924 | (25.1) |
| 200–400 | 2,374 | (44.0) | 5,101 | (43.7) |
| ≥400 | 1,753 | (32.5) | 3,377 | (29.0) |
| Unknown | 114 | (2.1) | 261 | (2.2) |
| **Marital status** |  |  |  |  |
| Living with spouse | 5,062 | (93.9) | 10,354 | (88.8) |
| Living alone | 321 | (6.0) | 1,299 | (11.1) |
| Unknown | 7 | (0.1) | 10 | (0.1) |
| **Current occupation** |  |  |  |  |
| Office | 1,956 | (36.3) | 1,903 | (16.3) |
| Manual | 2,421 | (44.9) | 3,124 | (26.8) |
| Unemployed/housewife | 934 | (17.3) | 6,554 | (56.2) |
| Soldier/etc. | 67 | (1.2) | 45 | (0.4) |
| Unknown | 12 | (0.2) | 37 | (0.3) |
| **BMI, kg/m^2^** |  |  |  |  |
| <18.5 | 65 | (1.2) | 289 | (2.5) |
| 18.5–23 | 1,609 | (29.9) | 5,320 | (45.6) |
| 23–25 | 1,673 | (31.0) | 3,032 | (26.0) |
| 25–30 | 1,914 | (35.5) | 2,755 | (23.6) |
| ≥30 | 129 | (2.4) | 267 | (2.3) |
| **Smoking** |  |  |  |  |
| Never | 1,662 | (30.8) | 11,364 | (97.4) |
| Former | 2,197 | (40.8) | 97 | (0.8) |
| Current | 1,527 | (28.3) | 193 | (1.7) |
| Unknown | 4 | (0.1) | 9 | (0.1) |
| **Drinking** |  |  |  |  |
| Never | 1,029 | (19.1) | 7,633 | (65.5) |
| Former | 343 | (6.4) | 169 | (1.5) |
| Current | 4,013 | (74.5) | 3,842 | (32.9) |
| Unknown | 5 | (0.1) | 19 | (0.2) |
| **Regular exercise,** Mean ± SD | 215.0 ± 299.8 | | 164.0 ± 230.6 | |
| No | 2,154 | (40.0) | 5,431 | (46.6) |
| Yes | 3,236 | (60.0) | 6,232 | (53.4) |

Supplementary Table S4. Distributions of biomarkers at baseline by sex

|  | Men | | | |  | Women | | | |
| --- | --- | --- | --- | --- | --- | --- | --- | --- | --- |
|  | N=5,390 | | | |  | N=11,663 | | | |
|  | Mean | (SD) | Median | (Q1–Q3) |  | Mean | (SD) | Median | (Q1–Q3) |
| Pulse (beats/minute) | 69.95 | (9.13) | 69.00 | (64.00–75.00) |  | 70.83 | (8.73) | 70.00 | (65.00–76.00) |
| SBP (mmHg) | 124.80 | (12.73) | 125.00 | (116.50–132.50) |  | 119.80 | (13.66) | 120.00 | (110.00–130.00) |
| DBP (mmHg) | 77.71 | (8.65) | 78.50 | (71.00–83.00) |  | 73.68 | (8.98) | 73.00 | (68.00–80.00) |
| Waist circumference (cm) | 85.06 | (7.43) | 85.00 | (80.00–90.00) |  | 77.28 | (8.02) | 77.00 | (71.80–82.50) |
| Hip circumference (cm) | 95.66 | (5.64) | 95.50 | (92.00–99.00) |  | 93.04 | (5.72) | 93.00 | (89.50–96.50) |
| Waist hip ratio | 0.89 | (0.05) | 0.89 | (0.86–0.92) |  | 0.83 | (0.06) | 0.83 | (0.79–0.87) |
| Body fat mass (kg) | 16.40 | (4.79) | 16.30 | (13.30–19.40) |  | 17.60 | (4.50) | 17.20 | (14.50–20.20) |
| Body fat percentage (%) | 23.26 | (4.44) | 23.60 | (20.60–26.30) |  | 30.20 | (4.29) | 30.30 | (27.40–33.10) |
| Visceral fat mass (kg) | 52.88 | (5.44) | 52.50 | (49.20–56.20) |  | 39.85 | (3.89) | 39.60 | (37.30–42.20) |
| Lean body mass (kg) | 48.92 | (4.95) | 48.50 | (45.50–51.90) |  | 36.59 | (3.49) | 36.40 | (34.30–38.70) |
| Muscle mass (kg) | 35.78 | (3.61) | 35.50 | (33.30–38.00) |  | 26.58 | (2.55) | 26.40 | (24.90–28.10) |
| Cell mass (kg) | 10.82 | (1.08) | 10.70 | (10.10–11.50) |  | 7.89 | (0.73) | 7.90 | (7.40–8.30) |
| Protein mass (kg) | 3.99 | (0.51) | 4.00 | (3.60–4.30) |  | 3.27 | (0.42) | 3.20 | (3.00–3.50) |
| Mineral mass (kg) | 2.47 | (0.95) | 2.40 | (1.80–3.00) |  | 2.03 | (0.81) | 1.90 | (1.50–2.40) |
| BUN (mg/dL) | 15.12 | (3.85) | 15.00 | (12.40–17.00) |  | 13.88 | (3.69) | 13.20 | (11.00–16.00) |
| Creatinine (mg/dL) | 0.94 | (0.16) | 0.93 | (0.86–1.01) |  | 0.71 | (0.14) | 0.70 | (0.63–0.77) |
| Uric acid (mg/dL) | 5.63 | (1.22) | 5.60 | (4.80–6.40) |  | 4.12 | (0.90) | 4.10 | (3.50–4.70) |
| Total cholesterol (mg/dL) | 193.37 | (33.19) | 192.00 | (172.00–214.00) |  | 199.64 | (33.83) | 197.00 | (176.00–221.00) |
| HDL (mg/dL) | 49.96 | (11.81) | 48.00 | (41.00–57.00) |  | 57.07 | (13.08) | 56.00 | (48.00–65.00) |
| LDL (mg/dL) | 114.09 | (30.81) | 113.60 | (94.40–133.20) |  | 120.93 | (30.66) | 118.60 | (99.60–140.20) |
| Triglyceride (mg/dL) | 146.61 | (102.39) | 120.00 | (83.00–177.00) |  | 108.23 | (69.15) | 92.00 | (65.00–132.00) |
| Fasting blood sugar (mg/dL) | 95.58 | (15.19) | 93.00 | (87.00–101.00) |  | 90.75 | (13.26) | 89.00 | (84.00–95.00) |
| HbA1c (%) | 5.65 | (0.58) | 5.60 | (5.30–5.80) |  | 5.60 | (0.51) | 5.50 | (5.30–5.80) |
| Albumin (g/dL) | 4.69 | (0.25) | 4.70 | (4.50–4.90) |  | 4.61 | (0.24) | 4.60 | (4.50–4.80) |
| AST (IU/L) | 24.65 | (12.00) | 22.00 | (19.00–27.00) |  | 21.72 | (9.14) | 20.00 | (17.00–23.00) |
| ALT (IU/L) | 25.50 | (16.65) | 21.00 | (16.00–29.00) |  | 18.84 | (13.20) | 16.00 | (13.00–21.00) |
| ALP (IU/L) | 207.90 | (76.13) | 209.00 | (173.00–250.00) |  | 194.49 | (84.61) | 193.00 | (146.00–245.00) |
| γ-GTP (IU/L) | 44.07 | (50.91) | 30.00 | (21.00–48.00) |  | 21.64 | (20.43) | 17.00 | (13.00–23.00) |
| Total bilirubin (mg/dL) | 0.86 | (0.33) | 0.80 | (0.60–1.00) |  | 0.69 | (0.25) | 0.70 | (0.50–0.80) |
| Direct bilirubin (mg/dL) | 0.28 | (0.11) | 0.30 | (0.20–0.30) |  | 0.21 | (0.08) | 0.20 | (0.20–0.30) |
| Indirect bilirubin (mg/dL) | 0.58 | (0.24) | 0.50 | (0.40–0.70) |  | 0.48 | (0.18) | 0.50 | (0.40–0.60) |
| Red blood cell count (million/µL) | 4.90 | (0.36) | 4.90 | (4.67–5.13) |  | 4.39 | (0.31) | 4.38 | (4.18–4.59) |
| Hemoglobin (g/dL) | 15.30 | (1.09) | 15.30 | (14.70–16.00) |  | 13.22 | (1.11) | 13.30 | (12.70–13.90) |
| Hematocrit (%) | 45.15 | (2.95) | 45.20 | (43.30–47.10) |  | 39.87 | (2.86) | 40.00 | (38.30–41.70) |
| MCV (fL) | 92.3 | (4.15) | 92.20 | (89.80–94.80) |  | 91.01 | (5.06) | 91.50 | (88.90–94.00) |
| MCH (pg) | 31.28 | (1.53) | 31.30 | (30.40–32.20) |  | 30.17 | (2.10) | 30.50 | (29.50–31.30) |
| MCHC (g/dL) | 33.89 | (0.89) | 33.90 | (33.30–34.40) |  | 33.13 | (1.05) | 33.20 | (32.60–33.80) |
| Platelet count (thousand/µL) | 237.67 | (52.46) | 233.00 | (203.00–269.00) |  | 261.47 | (58.93) | 257.00 | (223.00–296.00) |
| CRP (mg/dL) | 0.15 | (0.32) | 0.08 | (0.05–0.14) |  | 0.13 | (0.36) | 0.06 | (0.04–0.10) |
| White blood cell count (thousand/µL) | 6.03 | (1.57) | 5.80 | (4.94–6.87) |  | 5.43 | (1.42) | 5.25 | (4.46–6.19) |
| Grip strength (kg) | 36.72 | (8.40) | 36.80 | (32.00–41.80) |  | 22.2 | (5.32) | 22.35 | (19.20–25.30) |
| Calcium level (mg/dL) | 9.20 | (0.37) | 9.20 | (8.90–9.40) |  | 9.15 | (0.39) | 9.10 | (8.90–9.40) |

SBP: systolic blood pressure, DBP: diastolic blood pressure, BUN: blood urea nitrogen, HDL: high density lipoprotein-cholesterol, LDL: low density lipoprotein-cholesterol, HbA1c: hemoglobin A1c, AST: aspartate aminotransferase, ALT: alanine aminotransferase, ALP: alkaline phosphatase, γ-GTP: γ-glutamyl transpeptidase, MCV: mean corpuscular volume, MCH: mean corpuscular hemoglobin, MCHC: mean corpuscular hemoglobin concentration, CRP: C-reactive protein

Supplementary Table S5. Associations between participation in regular exercise and biomarkers at baseline

|  |  | Men | | | |  | Women | | | |  |  |
| --- | --- | --- | --- | --- | --- | --- | --- | --- | --- | --- | --- | --- |
| Class | Markers | beta | SE | p | fdr.adj |  | beta | SE | p | fdr.adj |  | p for heterogeneity |
| Blood pressure | Pulse | -0.1584 | 0.0286 | <0.0001 | <0.0001 |  | -0.1088 | 0.0188 | <0.0001 | <0.0001 |  | 0.1473 |
|  | SBP | 0.0090 | 0.0278 | 0.7449 | 0.8233 |  | 0.0318 | 0.0178 | 0.0750 | 0.1086 |  | 0.4898 |
|  | DBP | 0.0056 | 0.0278 | 0.8405 | 0.8472 |  | 0.0136 | 0.0183 | 0.4586 | 0.5351 |  | 0.8100 |
| Obesity-related | Waist circumference | -0.1032 | 0.0178 | <0.0001 | <0.0001 |  | -0.0735 | 0.0115 | <0.0001 | <0.0001 |  | 0.1611 |
|  | Hip circumference | -0.0050 | 0.0202 | 0.8036 | 0.8472 |  | -0.0523 | 0.0133 | 0.0001 | 0.0002 |  | 0.0505 |
|  | Waist hip ratio | -0.1564 | 0.0247 | <0.0001 | <0.0001 |  | -0.0603 | 0.0157 | 0.0001 | 0.0003 |  | 0.0010 |
|  | Body fat mass | -0.0261 | 0.0135 | 0.0534 | 0.0921 |  | 0.0025 | 0.0065 | 0.6986 | 0.7722 |  | 0.0563 |
|  | Body fat percentage | -0.0511 | 0.0174 | 0.0032 | 0.0097 |  | -0.0357 | 0.0081 | <0.0001 | <0.0001 |  | 0.4223 |
|  | Visceral fat mass | -0.0270 | 0.0141 | 0.0556 | 0.0921 |  | -0.0128 | 0.0067 | 0.0538 | 0.0807 |  | 0.3630 |
| Body composition | Lean body mass | 0.0689 | 0.0209 | 0.0010 | 0.0037 |  | 0.0779 | 0.0145 | <0.0001 | <0.0001 |  | 0.7235 |
|  | Muscle mass | 0.0751 | 0.0213 | 0.0004 | 0.0022 |  | 0.0808 | 0.0149 | <0.0001 | <0.0001 |  | 0.8264 |
|  | Cell mass | 0.0808 | 0.0211 | 0.0001 | 0.0011 |  | 0.0785 | 0.0150 | <0.0001 | <0.0001 |  | 0.9292 |
|  | Protein mass | 0.0846 | 0.0232 | 0.0003 | 0.0016 |  | 0.0900 | 0.0163 | <0.0001 | <0.0001 |  | 0.8490 |
|  | Mineral mass | 0.0384 | 0.0153 | 0.0118 | 0.0291 |  | 0.0482 | 0.0103 | <0.0001 | <0.0001 |  | 0.5952 |
| Renal function | BUN | 0.0607 | 0.0281 | 0.0310 | 0.0592 |  | 0.0643 | 0.0179 | 0.0003 | 0.0007 |  | 0.9140 |
|  | Creatinine | 0.0765 | 0.0282 | 0.0067 | 0.0176 |  | 0.1135 | 0.0189 | <0.0001 | <0.0001 |  | 0.2758 |
|  | Uric acid | 0.0130 | 0.0282 | 0.6446 | 0.7458 |  | 0.0428 | 0.0182 | 0.0189 | 0.0294 |  | 0.3746 |
| Lipids | Total cholesterol | -0.0127 | 0.0286 | 0.6570 | 0.7458 |  | -0.0043 | 0.0184 | 0.8131 | 0.8613 |  | 0.8049 |
|  | HDL | 0.0944 | 0.0274 | 0.0006 | 0.0027 |  | 0.0994 | 0.0182 | <0.0001 | <0.0001 |  | 0.8792 |
|  | LDL | 0.0070 | 0.0288 | 0.8076 | 0.8472 |  | -0.0016 | 0.0184 | 0.9309 | 0.9536 |  | 0.8013 |
|  | Triglyceride | -0.1076 | 0.0271 | 0.0001 | 0.0008 |  | -0.1095 | 0.0179 | <0.0001 | <0.0001 |  | 0.9533 |
| Glucose level | Fasting blood sugar | 0.0054 | 0.0282 | 0.8472 | 0.8472 |  | -0.0218 | 0.0184 | 0.2375 | 0.2881 |  | 0.4192 |
|  | HbA1c | -0.0619 | 0.0277 | 0.0253 | 0.0507 |  | -0.0208 | 0.0177 | 0.2401 | 0.2881 |  | 0.2112 |
| Liver function | Albumin | 0.0535 | 0.0281 | 0.0572 | 0.0921 |  | 0.0327 | 0.0188 | 0.0822 | 0.1114 |  | 0.5384 |
|  | AST | 0.0256 | 0.0284 | 0.3665 | 0.4527 |  | 0.0467 | 0.0180 | 0.0095 | 0.0160 |  | 0.5303 |
|  | ALT | -0.0493 | 0.0267 | 0.0649 | 0.0974 |  | -0.0245 | 0.0178 | 0.1679 | 0.2203 |  | 0.4396 |
|  | ALP | 0.0570 | 0.0286 | 0.0462 | 0.0843 |  | -0.0001 | 0.0177 | 0.9976 | 0.9976 |  | 0.0896 |
|  | γ-GTP | -0.0649 | 0.0264 | 0.0139 | 0.0308 |  | -0.0590 | 0.0181 | 0.0011 | 0.0022 |  | 0.8538 |
|  | Total bilirubin | 0.0895 | 0.0285 | 0.0017 | 0.0059 |  | 0.0917 | 0.0189 | <0.0001 | <0.0001 |  | 0.9487 |
|  | Direct bilirubin | 0.0438 | 0.0287 | 0.1267 | 0.1773 |  | 0.0468 | 0.0189 | 0.0132 | 0.0213 |  | 0.9304 |
|  | Indirect bilirubin | 0.0951 | 0.0285 | 0.0008 | 0.0035 |  | 0.0914 | 0.0189 | <0.0001 | <0.0001 |  | 0.9138 |
| Hematology | Red blood cell count | -0.0672 | 0.0273 | 0.0139 | 0.0308 |  | 0.0226 | 0.0188 | 0.2290 | 0.2881 |  | 0.0067 |
|  | Hemoglobin | -0.0350 | 0.0276 | 0.2055 | 0.2697 |  | 0.0529 | 0.0186 | 0.0045 | 0.0082 |  | 0.0083 |
|  | Hematocrit | -0.0225 | 0.0279 | 0.4192 | 0.5030 |  | 0.0632 | 0.0186 | 0.0007 | 0.0014 |  | 0.0106 |
|  | MCV | 0.0782 | 0.0273 | 0.0042 | 0.0116 |  | 0.0481 | 0.0184 | 0.0087 | 0.0153 |  | 0.3606 |
|  | MCH | 0.0523 | 0.0277 | 0.0592 | 0.0921 |  | 0.0323 | 0.0185 | 0.0819 | 0.1114 |  | 0.5482 |
|  | MCHC | -0.0310 | 0.0286 | 0.2783 | 0.3542 |  | -0.0118 | 0.0189 | 0.5314 | 0.6033 |  | 0.5754 |
|  | Platelet count | -0.0440 | 0.0285 | 0.1224 | 0.1772 |  | -0.0568 | 0.0186 | 0.0023 | 0.0043 |  | 0.7068 |
| Inflammation | CRP | -0.0834 | 0.0278 | 0.0027 | 0.0087 |  | -0.0806 | 0.0177 | <0.0001 | <0.0001 |  | 0.9323 |
|  | White blood cell count | -0.0632 | 0.0275 | 0.0217 | 0.0456 |  | -0.0752 | 0.0186 | 0.0001 | 0.0001 |  | 0.7178 |
| Others | Grip strength | 0.0996 | 0.0272 | 0.0003 | 0.0016 |  | 0.1085 | 0.0181 | <0.0001 | <0.0001 |  | 0.7853 |
|  | Calcium level | 0.0398 | 0.0287 | 0.1649 | 0.2235 |  | -0.0042 | 0.0184 | 0.8203 | 0.8613 |  | 0.1968 |

Adjusted for age, education level, income, marital status, occupation, smoking status, drinking status, and BMI (+ menopause for women)

SBP: systolic blood pressure, DBP: diastolic blood pressure, BUN: blood urea nitrogen, HDL: high density lipoprotein-cholesterol, LDL: low density lipoprotein-cholesterol, HbA1c: hemoglobin A1c, AST: aspartate aminotransferase, ALT: alanine aminotransferase, ALP: alkaline phosphatase, γ-GTP: γ-glutamyl transpeptidase, MCV: mean corpuscular volume, MCH: mean corpuscular hemoglobin, MCHC: mean corpuscular hemoglobin concentration, CRP: C-reactive protein

Supplementary Table S6. Associations between participation in regular exercise and risk of each cardiovascular & metabolic disease

|  | Men | | | |  |  |  | Women | | | |  |  |
| --- | --- | --- | --- | --- | --- | --- | --- | --- | --- | --- | --- | --- | --- |
|  | No. of cases | | | |  |  |  | No. of cases | | | |  |  |
| Diseases | Nonparticipants (N=2,154) | | Participants (N=3,236) | | HR^a^ | (95% CI) |  | Nonparticipants (N=5,431) | | Participants (N=6,232) | | HR^a^ | (95% CI) |
| Diabetes | 80 | (3.7) | 95 | (2.9) | 0.83 | (0.60-1.16) |  | 124 | (2.3) | 106 | (1.7) | 0.77 | (0.58-1.03) |
| Hypertension | 153 | (7.1) | 212 | (6.6) | 0.96 | (0.76-1.21) |  | 270 | (5.0) | 312 | (5.0) | 1.01 | (0.84-1.20) |
| Dyslipidemia | 177 | (8.2) | 232 | (7.2) | 0.86 | (0.69-1.08) |  | 603 | (11.1) | 699 | (11.2) | 0.95 | (0.84-1.07) |
| Any cardiovascular & metabolic disease no. of disease ≥ 1 | 339 | (15.8) | 458 | (14.2) | 0.88 | (0.75-1.03) |  | 866 | (16.0) | 993 | (16.0) | 0.95 | (0.86-1.05) |
| no. of disease ≥ 2 | 63 | (3.4) | 70 | (2.5) | 0.73 | (0.49-1.08) |  | 121 | (2.6) | 119 | (2.2) | 0.88 | (0.66-1.18) |

^a^ Age as a time scale and adjusted for education level, income, marital status, occupation, smoking status, drinking status, BMI**,** and diseases reciprocally (+menopause status for women)

Supplementary Table S7. Associations between biomarkers and the risk of each cardiovascular & metabolic disease

(A) Men

|  |  | Diabetes | |  | Hypertension | |  | Dyslipidemia | |  | No. of disease ≥ 1 | |  | No. of disease ≥ 2 | |
| --- | --- | --- | --- | --- | --- | --- | --- | --- | --- | --- | --- | --- | --- | --- | --- |
| Class | Markers | HR | (95% CI) |  | HR | (95% CI) |  | HR | (95% CI) |  | HR | (95% CI) |  | HR | (95% CI) |
| Blood pressure | Pulse | 1.25 | (1.07–1.46) |  | 1.06 | (0.95–1.19) |  | 0.94 | (0.85–1.05) |  | 1.06 | (0.98–1.15) |  | 1.02 | (0.84–1.24) |
|  | SBP | 1.08 | (0.91–1.29) |  | 2.96 | (2.63–3.34) |  | 0.91 | (0.81–1.01) |  | 1.60 | (1.47–1.73) |  | 2.29 | (1.86–2.82) |
|  | DBP | 1.03 | (0.86–1.22) |  | 2.44 | (2.16–2.74) |  | 0.94 | (0.84–1.06) |  | 1.48 | (1.37–1.61) |  | 1.77 | (1.45–2.17) |
| Obesity-related | Waist circumference | 1.47 | (1.13–1.91) |  | 0.99 | (0.83–1.18) |  | 1.11 | (0.93–1.32) |  | 1.13 | (1.00–1.28) |  | 1.25 | (0.92–1.71) |
|  | Hip circumference | 1.02 | (0.81–1.29) |  | 0.84 | (0.71–0.98) |  | 1.00 | (0.86–1.17) |  | 0.90 | (0.81–1.01) |  | 0.94 | (0.71–1.23) |
|  | Waist hip ratio | 1.36 | (1.13–1.64) |  | 1.13 | (0.99–1.29) |  | 1.07 | (0.95–1.22) |  | 1.18 | (1.08–1.29) |  | 1.25 | (1.00–1.56) |
|  | Body fat mass | 1.51 | (1.08–2.10) |  | 0.93 | (0.74–1.17) |  | 1.06 | (0.84–1.33) |  | 1.05 | (0.89–1.23) |  | 1.43 | (0.95–2.15) |
|  | Body fat percentage | 1.33 | (1.03–1.72) |  | 1.01 | (0.84–1.20) |  | 1.08 | (0.91–1.28) |  | 1.07 | (0.95–1.21) |  | 1.46 | (1.06–1.99) |
|  | Visceral fat mass | 1.58 | (1.14–2.20) |  | 0.94 | (0.76–1.17) |  | 1.02 | (0.82–1.26) |  | 1.05 | (0.90–1.22) |  | 1.40 | (0.95–2.06) |
| Body composition | Lean body mass | 0.97 | (0.77–1.20) |  | 0.84 | (0.72–0.98) |  | 0.88 | (0.76–1.01) |  | 0.86 | (0.78–0.96) |  | 0.76 | (0.59–0.98) |
|  | Muscle mass | 1.00 | (0.80–1.24) |  | 0.86 | (0.74–0.99) |  | 0.86 | (0.74–0.99) |  | 0.88 | (0.79–0.97) |  | 0.75 | (0.59–0.97) |
|  | Cell mass | 1.03 | (0.82–1.27) |  | 0.86 | (0.74–1.00) |  | 0.86 | (0.75–1.00) |  | 0.88 | (0.80–0.98) |  | 0.77 | (0.60–0.99) |
|  | Protein mass | 0.97 | (0.80–1.18) |  | 0.87 | (0.76–1.00) |  | 0.87 | (0.77–0.99) |  | 0.89 | (0.81–0.97) |  | 0.77 | (0.61–0.96) |
|  | Mineral mass | 1.17 | (0.86–1.59) |  | 0.82 | (0.67–1.01) |  | 0.81 | (0.67–0.99) |  | 0.85 | (0.74–0.98) |  | 0.76 | (0.54–1.09) |
| Renal function | BUN | 1.09 | (0.92–1.28) |  | 0.98 | (0.87–1.09) |  | 1.01 | (0.90–1.12) |  | 1.02 | (0.94–1.10) |  | 1.00 | (0.83–1.21) |
|  | Creatinine | 0.95 | (0.81–1.11) |  | 0.93 | (0.83–1.05) |  | 1.05 | (0.94–1.17) |  | 0.98 | (0.91–1.06) |  | 0.95 | (0.78–1.15) |
|  | Uric acid | 0.92 | (0.79–1.08) |  | 1.01 | (0.90–1.12) |  | 1.11 | (1.00–1.24) |  | 1.03 | (0.95–1.11) |  | 1.08 | (0.89–1.30) |
| Lipids | Total cholesterol | 1.08 | (0.92–1.27) |  | 1.15 | (1.03–1.29) |  | 1.79 | (1.61–1.99) |  | 1.44 | (1.33–1.55) |  | 1.83 | (1.51–2.22) |
|  | HDL | 0.90 | (0.76–1.05) |  | 1.11 | (0.98–1.24) |  | 0.91 | (0.82–1.02) |  | 0.96 | (0.89–1.04) |  | 0.88 | (0.72–1.08) |
|  | LDL | 0.99 | (0.85–1.15) |  | 1.09 | (0.97–1.21) |  | 1.42 | (1.27–1.57) |  | 1.23 | (1.14–1.33) |  | 1.38 | (1.14–1.66) |
|  | Triglyceride | 1.38 | (1.16–1.64) |  | 1.04 | (0.92–1.17) |  | 1.58 | (1.41–1.77) |  | 1.35 | (1.25–1.47) |  | 1.91 | (1.55–2.34) |
| Glucose level | Fasting blood sugar | 4.73 | (3.93–5.69) |  | 0.94 | (0.84–1.06) |  | 1.01 | (0.90–1.12) |  | 1.34 | (1.24–1.45) |  | 2.23 | (1.82–2.73) |
|  | HbA1c | 5.59 | (4.64–6.73) |  | 0.91 | (0.81–1.03) |  | 1.15 | (1.02–1.29) |  | 1.43 | (1.32–1.55) |  | 2.85 | (2.31–3.51) |
| Liver function | Albumin | 1.09 | (0.93–1.27) |  | 1.15 | (1.02–1.28) |  | 1.18 | (1.06–1.32) |  | 1.18 | (1.09–1.28) |  | 1.28 | (1.05–1.56) |
|  | AST | 1.08 | (0.92–1.27) |  | 1.20 | (1.08–1.35) |  | 1.03 | (0.93–1.15) |  | 1.10 | (1.02–1.19) |  | 1.39 | (1.15–1.69) |
|  | ALT | 1.46 | (1.22–1.75) |  | 1.16 | (1.03–1.32) |  | 1.15 | (1.02–1.29) |  | 1.21 | (1.11–1.31) |  | 1.75 | (1.42–2.14) |
|  | ALP | 1.26 | (1.06–1.49) |  | 1.08 | (0.96–1.22) |  | 1.06 | (0.95–1.19) |  | 1.13 | (1.04–1.22) |  | 1.41 | (1.16–1.73) |
|  | γ-GTP | 1.49 | (1.25–1.79) |  | 1.27 | (1.12–1.44) |  | 1.21 | (1.07–1.36) |  | 1.29 | (1.19–1.41) |  | 1.95 | (1.59–2.40) |
|  | Total bilirubin | 1.04 | (0.88–1.23) |  | 1.04 | (0.92–1.17) |  | 0.95 | (0.85–1.06) |  | 0.99 | (0.92–1.07) |  | 1.07 | (0.88–1.30) |
|  | Direct bilirubin | 1.05 | (0.89–1.24) |  | 1.00 | (0.90–1.13) |  | 0.82 | (0.73–0.91) |  | 0.91 | (0.84–0.98) |  | 0.90 | (0.74–1.09) |
|  | Indirect bilirubin | 1.03 | (0.87–1.22) |  | 1.06 | (0.95–1.19) |  | 1.03 | (0.92–1.14) |  | 1.04 | (0.96–1.12) |  | 1.14 | (0.94–1.38) |
| Hematology | Red blood cell count | 1.33 | (1.13–1.57) |  | 1.12 | (1.00–1.26) |  | 1.11 | (0.99–1.24) |  | 1.17 | (1.08–1.27) |  | 1.53 | (1.26–1.86) |
|  | Hemoglobin | 1.22 | (1.04–1.44) |  | 1.19 | (1.06–1.33) |  | 1.07 | (0.96–1.19) |  | 1.17 | (1.08–1.26) |  | 1.47 | (1.21–1.78) |
|  | Hematocrit | 1.16 | (0.98–1.37) |  | 1.18 | (1.05–1.32) |  | 1.09 | (0.98–1.22) |  | 1.15 | (1.07–1.25) |  | 1.49 | (1.23–1.80) |
|  | MCV | 0.79 | (0.67–0.93) |  | 1.07 | (0.95–1.20) |  | 0.96 | (0.86–1.07) |  | 0.96 | (0.89–1.04) |  | 0.92 | (0.76–1.12) |
|  | MCH | 0.89 | (0.75–1.06) |  | 1.08 | (0.96–1.22) |  | 0.94 | (0.84–1.05) |  | 0.99 | (0.92–1.07) |  | 0.95 | (0.78–1.15) |
|  | MCHC | 1.18 | (1.00–1.38) |  | 1.03 | (0.93–1.16) |  | 0.98 | (0.88–1.09) |  | 1.06 | (0.98–1.15) |  | 1.04 | (0.86–1.26) |
|  | Platelet count | 1.06 | (0.90–1.24) |  | 0.95 | (0.85–1.06) |  | 1.13 | (1.01–1.25) |  | 1.02 | (0.95–1.10) |  | 1.13 | (0.94–1.37) |
| Inflammation | CRP | 1.42 | (1.19–1.68) |  | 1.23 | (1.09–1.38) |  | 1.09 | (0.97–1.22) |  | 1.21 | (1.12–1.31) |  | 1.56 | (1.27–1.92) |
|  | White blood cell count | 1.56 | (1.31–1.86) |  | 1.03 | (0.92–1.16) |  | 1.12 | (1.00–1.26) |  | 1.17 | (1.08–1.27) |  | 1.47 | (1.20–1.79) |
| Others | Grip strength | 1.02 | (0.87–1.21) |  | 0.94 | (0.84–1.06) |  | 1.01 | (0.90–1.13) |  | 1.00 | (0.93–1.09) |  | 0.86 | (0.71–1.04) |
|  | Calcium level | 1.10 | (0.94–1.29) |  | 1.03 | (0.92–1.15) |  | 1.15 | (1.03–1.28) |  | 1.09 | (1.01–1.17) |  | 1.23 | (1.02–1.49) |

Age as time scale and adjusted for education level, income, marital status, occupation, smoking status, drinking status, BMI, and diseases (diabetes, hypertension, and dyslipidemia, reciprocally)

SBP: systolic blood pressure, DBP: diastolic blood pressure, BUN: blood urea nitrogen, HDL: high density lipoprotein-cholesterol, LDL: low density lipoprotein-cholesterol, HbA1c: hemoglobin A1c, AST: aspartate aminotransferase, ALT: alanine aminotransferase, ALP: alkaline phosphatase, γ-GTP: γ-glutamyl transpeptidase, MCV: mean corpuscular volume, MCH: mean corpuscular hemoglobin, MCHC: mean corpuscular hemoglobin concentration, CRP: C-reactive protein

(B) Women

|  |  | Diabetes | |  | Hypertension | |  | Dyslipidemia | |  | no. of disease ≥ 1 | |  | no. of disease ≥ 2 | |
| --- | --- | --- | --- | --- | --- | --- | --- | --- | --- | --- | --- | --- | --- | --- | --- |
| Class | Markers | HR | (95% CI) |  | HR | (95% CI) |  | HR | (95% CI) |  | HR | (95% CI) |  | HR | (95% CI) |
| Hypertension | Pulse | 1.10 | (0.96–1.27) |  | 1.10 | (1.00–1.20) |  | 1.03 | (0.97–1.09) |  | 1.06 | (1.01–1.11) |  | 1.11 | (0.96–1.29) |
|  | SBP | 1.23 | (1.04–1.45) |  | 3.71 | (3.36–4.10) |  | 1.03 | (0.96–1.10) |  | 1.51 | (1.43–1.60) |  | 3.01 | (2.55–3.54) |
|  | DBP | 1.35 | (1.15–1.57) |  | 3.32 | (3.01–3.66) |  | 1.03 | (0.96–1.09) |  | 1.49 | (1.42–1.57) |  | 2.74 | (2.34–3.20) |
| Obesity | Waist circumference | 1.53 | (1.19–1.96) |  | 1.12 | (0.96–1.30) |  | 1.05 | (0.96–1.16) |  | 1.11 | (1.02–1.20) |  | 1.28 | (1.00–1.63) |
|  | Hip circumference | 0.89 | (0.72–1.09) |  | 0.93 | (0.82–1.06) |  | 0.83 | (0.76–0.90) |  | 0.87 | (0.81–0.93) |  | 0.71 | (0.58–0.87) |
|  | Waist hip ratio | 1.44 | (1.22–1.72) |  | 1.13 | (1.02–1.26) |  | 1.15 | (1.07–1.24) |  | 1.17 | (1.10–1.24) |  | 1.49 | (1.25–1.78) |
|  | Body fat mass | 1.59 | (1.03–2.44) |  | 1.02 | (0.78–1.32) |  | 0.99 | (0.83–1.18) |  | 1.03 | (0.89–1.20) |  | 1.18 | (0.77–1.82) |
|  | Body fat percentage | 1.24 | (0.89–1.73) |  | 0.97 | (0.79–1.19) |  | 1.15 | (1.00–1.33) |  | 1.10 | (0.98–1.24) |  | 1.22 | (0.87–1.72) |
|  | Visceral fat mass | 1.48 | (0.96–2.27) |  | 0.94 | (0.73–1.21) |  | 1.02 | (0.86–1.21) |  | 1.02 | (0.88–1.17) |  | 1.18 | (0.77–1.79) |
| Body composition | Lean body mass | 1.09 | (0.90–1.31) |  | 1.05 | (0.93–1.17) |  | 0.89 | (0.82–0.96) |  | 0.95 | (0.89–1.01) |  | 0.95 | (0.79–1.15) |
|  | Muscle mass | 1.08 | (0.90–1.29) |  | 1.04 | (0.94–1.17) |  | 0.89 | (0.82–0.95) |  | 0.94 | (0.89–1.01) |  | 0.95 | (0.80–1.14) |
|  | Cell mass | 1.12 | (0.94–1.34) |  | 1.03 | (0.92–1.14) |  | 0.90 | (0.84–0.97) |  | 0.95 | (0.90–1.01) |  | 0.97 | (0.82–1.16) |
|  | Protein mass | 1.06 | (0.90–1.25) |  | 1.04 | (0.94–1.15) |  | 0.89 | (0.84–0.96) |  | 0.95 | (0.89–1.00) |  | 0.95 | (0.81–1.12) |
|  | Mineral mass | 1.19 | (0.91–1.56) |  | 1.03 | (0.88–1.22) |  | 0.86 | (0.77–0.96) |  | 0.93 | (0.85–1.02) |  | 0.91 | (0.70–1.19) |
| Renal function | BUN | 1.06 | (0.91–1.23) |  | 0.97 | (0.89–1.07) |  | 0.99 | (0.94–1.06) |  | 0.99 | (0.94–1.04) |  | 1.04 | (0.89–1.21) |
|  | Creatinine | 0.92 | (0.80–1.06) |  | 1.02 | (0.93–1.11) |  | 0.99 | (0.94–1.05) |  | 0.99 | (0.94–1.04) |  | 0.98 | (0.85–1.13) |
|  | Uric acid | 1.08 | (0.93–1.24) |  | 1.11 | (1.01–1.21) |  | 1.11 | (1.04–1.18) |  | 1.11 | (1.06–1.17) |  | 1.18 | (1.02–1.36) |
| Dyslipidemia | Total cholesterol | 1.16 | (1.00–1.35) |  | 1.01 | (0.92–1.10) |  | 2.00 | (1.88–2.13) |  | 1.62 | (1.54–1.71) |  | 2.07 | (1.78–2.41) |
|  | HDL | 0.74 | (0.63–0.86) |  | 0.94 | (0.86–1.03) |  | 0.92 | (0.87–0.98) |  | 0.89 | (0.85–0.94) |  | 0.85 | (0.73–0.99) |
|  | LDL | 0.95 | (0.83–1.10) |  | 1.01 | (0.92–1.10) |  | 1.88 | (1.76–2.00) |  | 1.54 | (1.46–1.62) |  | 1.71 | (1.47–1.99) |
|  | Triglyceride | 1.74 | (1.49–2.02) |  | 1.10 | (1.00–1.21) |  | 1.44 | (1.35–1.54) |  | 1.38 | (1.31–1.46) |  | 1.91 | (1.64–2.22) |
| Diabetes | Fasting blood sugar | 5.44 | (4.58–6.47) |  | 1.01 | (0.92–1.11) |  | 1.10 | (1.03–1.16) |  | 1.28 | (1.21–1.34) |  | 1.81 | (1.55–2.11) |
|  | HbA1c | 7.33 | (6.10–8.82) |  | 0.97 | (0.88–1.07) |  | 1.19 | (1.12–1.28) |  | 1.37 | (1.30–1.45) |  | 2.09 | (1.78–2.45) |
| Liver function | Albumin | 1.15 | (1.00–1.33) |  | 1.01 | (0.93–1.10) |  | 1.10 | (1.04–1.16) |  | 1.11 | (1.06–1.17) |  | 1.06 | (0.91–1.22) |
|  | AST | 1.37 | (1.19–1.59) |  | 0.98 | (0.89–1.07) |  | 1.01 | (0.95–1.08) |  | 1.03 | (0.98–1.09) |  | 1.21 | (1.04–1.40) |
|  | ALT | 1.97 | (1.69–2.30) |  | 1.00 | (0.91–1.10) |  | 1.13 | (1.06–1.20) |  | 1.16 | (1.10–1.22) |  | 1.51 | (1.30–1.76) |
|  | ALP | 1.45 | (1.23–1.72) |  | 1.25 | (1.12–1.38) |  | 1.05 | (0.98–1.12) |  | 1.16 | (1.09–1.22) |  | 1.36 | (1.16–1.61) |
|  | γ-GTP | 1.96 | (1.68–2.28) |  | 1.10 | (1.00–1.20) |  | 1.14 | (1.07–1.21) |  | 1.20 | (1.13–1.26) |  | 1.58 | (1.36–1.84) |
|  | Total bilirubin | 0.96 | (0.83–1.11) |  | 1.05 | (0.96–1.15) |  | 0.98 | (0.92–1.04) |  | 1.00 | (0.95–1.05) |  | 1.00 | (0.86–1.15) |
|  | Direct bilirubin | 0.96 | (0.83–1.10) |  | 1.04 | (0.96–1.14) |  | 0.82 | (0.78–0.87) |  | 0.88 | (0.83–0.92) |  | 0.87 | (0.75–1.01) |
|  | Indirect bilirubin | 0.97 | (0.84–1.12) |  | 1.05 | (0.96–1.14) |  | 1.07 | (1.00–1.13) |  | 1.06 | (1.01–1.11) |  | 1.05 | (0.91–1.22) |
| Hematology | Red blood cell count | 1.75 | (1.51–2.02) |  | 1.26 | (1.15–1.38) |  | 1.09 | (1.03–1.15) |  | 1.20 | (1.14–1.26) |  | 1.51 | (1.31–1.75) |
|  | Hemoglobin | 1.52 | (1.31–1.75) |  | 1.15 | (1.05–1.26) |  | 1.07 | (1.01–1.14) |  | 1.15 | (1.09–1.21) |  | 1.38 | (1.19–1.60) |
|  | Hematocrit | 1.48 | (1.28–1.71) |  | 1.18 | (1.08–1.29) |  | 1.07 | (1.01–1.14) |  | 1.14 | (1.09–1.20) |  | 1.40 | (1.21–1.61) |
|  | MCV | 0.77 | (0.66–0.90) |  | 0.92 | (0.84–1.01) |  | 0.96 | (0.90–1.02) |  | 0.92 | (0.87–0.97) |  | 0.85 | (0.73–0.99) |
|  | MCH | 0.87 | (0.75–1.01) |  | 0.92 | (0.84–1.01) |  | 0.97 | (0.91–1.03) |  | 0.94 | (0.89–0.99) |  | 0.91 | (0.78–1.06) |
|  | MCHC | 1.13 | (0.98–1.31) |  | 0.93 | (0.85–1.01) |  | 1.04 | (0.98–1.11) |  | 1.03 | (0.98–1.08) |  | 1.03 | (0.89–1.19) |
|  | Platelet count | 1.04 | (0.90–1.20) |  | 1.07 | (0.98–1.17) |  | 1.19 | (1.12–1.27) |  | 1.15 | (1.10–1.21) |  | 1.26 | (1.09–1.47) |
| Inflammation | CRP | 1.53 | (1.31–1.80) |  | 1.14 | (1.03–1.26) |  | 1.09 | (1.02–1.17) |  | 1.15 | (1.09–1.22) |  | 1.35 | (1.15–1.58) |
|  | White blood cell count | 1.43 | (1.24–1.65) |  | 0.98 | (0.90–1.07) |  | 1.06 | (1.00–1.13) |  | 1.07 | (1.01–1.12) |  | 1.26 | (1.09–1.45) |
| Others | Grip strength | 0.87 | (0.75–1.01) |  | 0.97 | (0.88–1.06) |  | 1.06 | (0.99–1.12) |  | 1.02 | (0.97–1.07) |  | 0.92 | (0.80–1.07) |
|  | Calcium level | 1.17 | (1.01–1.36) |  | 0.98 | (0.89–1.07) |  | 1.11 | (1.05–1.18) |  | 1.10 | (1.05–1.16) |  | 1.12 | (0.96–1.29) |

Age as time scale and adjusted for education level, income, marital status, occupation, smoking status, drinking status, BMI, menopause status, and diseases (diabetes, hypertension, and dyslipidemia, reciprocally)

SBP: systolic blood pressure, DBP: diastolic blood pressure, BUN: blood urea nitrogen, HDL: high density lipoprotein-cholesterol, LDL: low density lipoprotein-cholesterol, HbA1c: hemoglobin A1c, AST: aspartate aminotransferase, ALT: alanine aminotransferase, ALP: alkaline phosphatase, γ-GTP: γ-glutamyl transpeptidase, MCV: mean corpuscular volume, MCH: mean corpuscular hemoglobin, MCHC: mean corpuscular hemoglobin concentration, CRP: C-reactive protein
